# Epstein–Barr virus-encoded microRNA BART22 serves as novel biomarkers and drives malignant transformation of nasopharyngeal carcinoma

**DOI:** 10.1038/s41419-022-05107-x

**Published:** 2022-07-30

**Authors:** Ting Zhang, Zui Chen, Jing Deng, Kaixiong Xu, Di Che, Jiamin Lin, Ping Jiang, Xiaoqiong Gu, Banglao Xu

**Affiliations:** 1 Department of Laboratory Medicine, the Second Affiliated Hospital, School of Medicine, South China University of Technology, Guangzhou, 510180 China; 2 Department of Clinical Biological Resource Bank, Guangzhou Institute of Pediatrics, Guangzhou Women and Children’s Medical Center, Guangzhou Medical University, Guangzhou, China

**Keywords:** Oral cancer, Tumour virus infections

## Abstract

Nasopharyngeal carcinoma (NPC) is an epithelial malignancy ubiquitously associated with Epstein–Barr virus (EBV). EBV generates various viral microRNAs (miRNAs) by processing the BHRF1 and BamHI A rightward (BART) transcripts. These BART miRNAs are abundantly expressed in NPC, but their functions and molecular mechanisms remain largely unknown. Our study found that the EBV-encoded microRNA BART-22 was significantly upregulated in NPC tissues and positively correlated with tumor progression. Furthermore, we found that EBV-miR-BART-22 was a significant predictor of poor prognosis in NPC. A reliable nomogram model to predict the preoperative overall survival (OS) of NPC patients was established. The area under the receiver operating characteristic (ROC) curve value for 5-year survival was 0.91. Elevated levels of EBV-miR-BART-22 significantly promoted the epithelial-mesenchymal transition (EMT) and metastasis of NPC cells in vivo and in vitro. We found that EBV-miR-BART-22 directly targets the 3′-UTR of MOSPD2 mRNA to promote the EMT and metastasis of NPC cells by activating the Wnt/β-catenin signaling pathway. Our findings provide a potential prognostic biomarker and new insight into the molecular mechanisms of NPC metastasis.

## Introduction

Nasopharyngeal carcinoma (NPC) is a squamous cell carcinoma derived from the nasopharyngeal epithelium of the pharyngeal recess (fossa of Rosenmüller) of the nasal cavity [1]. It has received much attention because of its very uneven distribution around the globe. As a rare type of head and neck cancer, the NPC age-standardized annual rate in most parts of the world is <1 per 100,000. However, the annual incidence of nasopharyngeal cancer has increased to 25–30 occurrences per 100,000 individuals in southern China and Southeast Asia [2, 3]. Each year, ~129,000 new cases are reported worldwide, with >70% of cases reported in South China and Southeast Asia [4, 5]. NPC development is associated with a combination of different risk factors: Epstein–Barr virus (EBV) infection, gender and genetic, environmental, and dietary factors [6, 7]. NPC is a malignant tumor in the nasopharynx and is prone to distant metastasis at an early stage. NPC distant sites of metastasis included the liver, lung, spleen, pleura, and bone. Unfortunately, as many as 60–70% of NPC cases remain undetected until the advanced stage of the disease due to the unique anatomical sites and the absence of early symptoms [8, 9]. Therefore, it is urgent to explore the underlying mechanism of NPC metastasis and find new effective drug candidates against NPC metastasis.

Although metastasis is responsible for more than 90% of cancer-related deaths, it is still the most poorly understood mechanism in tumor etiology [10]. As the crucial step of cancer metastasis, epithelial-to-mesenchymal transition (EMT) has been shown to play a critical role in cancer cell metastatic dissemination events by endowing cancer cells with a more motile and invasive phenotype [11, 12]. Moreover, EMT improves the ability of the cell to resist damage by changing morphological structure and developing apoptosis resistance mechanisms, which are critical for cancer cell survival in extreme environments during the migration from the primary site of origin to distant sites [13]. According to previous studies, there are two central different cell states (epithelial and mesenchymal) in the process of EMT, which is usually characterized by the loss of the epithelial characteristics E-cadherin and α-catenin in cancer cells and the acquisition of the mesenchymal marker vimentin and N-cadherin, as well as imparting tumor cell metastasis [14]. Similarly, EMT is also closely related to the invasion and metastasis of NPC [15, 16]. Therefore, promoting EMT in NPC could significantly increase the metastatic ability of NPC.

The Epstein–Barr virus (EBV) discovered in 1964 is the first virus that was correlated with cancers in humans [17]. EBV potently infects most individuals worldwide and is etiologically related to several lymphoid and epithelial malignancies, including Burkitt’s lymphoma, leiomyosarcoma, B-cell lymphoma, NK, and T-cell lymphomas, gastric cancer, and nasopharyngeal cancer [18]. Tissue-specific EBV infections make EBV-related tumors usually express only limited virus-encoded proteins, which demonstrates that tumorigenesis and tumor progression are not only affected by EBV-encoded proteins but may also be influenced by other essential factors, such as EBV-encoded microRNAs (EBV-miRNAs) [19]. MiRNAs are a class of small RNAs that are ~17–24 nt in length and modulate gene expression by degrading mRNA or inhibiting translation [20]. Recent studies have shown that the BamHI fragment A rightward transcript region of EBV encodes 44 mature microRNAs, some of which are highly expressed in NPC [21, 22]. Previous studies have found that these EBV-miR-BART transcripts play a key role in the occurrence and development of NPC, including cell cycle regulation, cellular migration and invasion, activation of the EMT program, stemness, and regulation of immune responses [23, 24]. EBV-miR-BART1-5p, EBV-miR-BART5, EBV-miR-BART13, and EBV-miR-BART20-5p were reported to promote NPC cell metastasis by targeting different proteins [25–28]. Nevertheless, the role of EBV-miR-BART22 in the occurrence and development of NPC is still unclear.

Here we report that NPC patients with higher expression of EBV-miR-BART-22 in tissue tend to develop distant metastasis and have a shorter survival time. EBV-miR-BART-22 enhances the ability of cells to migrate and invade in vitro. Using the spleen injection metastasis model, we found that EBV-miR-BART-22 facilitated the development of more metastatic lesions. Finally, we discovered that EBV-miR-BART-22 could activate the Wnt/β-catenin signaling pathway by directly binding with MOSPD2. The findings of this study provide novel insights into NPC metastasis regulated by EBV.

## Methods

### Data collection and preprocessing

The miRNA expression datasets were downloaded from the GEO (ID: GSE54161, GSE118720). Normalized log-transformed array data for the transcriptome and GEO datasets were conducted by R software (version 4.0.2).

### RNA-deep sequencing

The RNA-seq experiments were performed and analyzed by Novogene. According to the manufacturer’s instruction, the DNA was removed from total RNAs by digesting in column with RNAse-free DNAse according to manufacturer’s instruction. The library and sequencing of transcriptome were prepared using Illumina HiSeq X Ten (Novogene Bioinformatics Technology Co., Ltd., Beijing, China). The mapping of 100-bp paired-end reads to genes was undertaken using HTSeq v0.6.0 software, while fragments per kilobase of transcript per million fragments mapped (FPKM) were also analyzed.

### Cell lines and culture

CNE-1, CNE-2, and SUNE-1 were EBV-negative NPC cell lines. C666-1 is an EBV-positive NPC cell line. CNE-1, CNE-2, SUNE-1, and C666-1 were cultured using RPIM 1640 medium (Gibco, Beijing, China) supplemented with 10% fetal bovine serum (FBS) (Gibco, Montevideo, Uruguay). HEK293T cells grew in Dulbecco’s modified Eagle’s medium (Gibco, Beijing, China) with 10% FBS.

### Ethics statement

The human tissue specimen research was approved by the institutional ethics committee of Shanghai Outdo Biotech Co, Shanghai, China (Exp. number: YB M-05-02). The animal research was approved by the Laboratory Animal Research Center, South China University of Technology (AEC: 2019030).

### RT-qPCR

The total RNA of NPC cell lines treated with Trizol reagent (Invitrogen, Shanghai, USA) and reverse transcription of microRNA was performed with the Mir-X miRNA First-Strand Synthesis Kit (TaKaRa Bio Inc, Dalian, China). The quantitative real-time PCR assay for EBV-miR-BART-22 was carried out using the TB Green Premix Ex Taq (TaKaRa Bio Inc, Dalian, China). U6 was served as normalizing the expression of miRNA. Total mRNA was extracted with Ultrapure RNA Kit (KangWei, Beijing, China), complementary DNA (cDNA) was synthesized with the PrimeScript RT reagent Kit (TaKaRa, Dalian, China). GAPDH or β-actin was served as normalizing the expression of mRNA, respectively. The fold changes were calculated using the 2^−ΔΔCt^ method. The sequences of real-time PCR primers used are listed in Table S5.

### Western blot analysis

Western blotting analyses were performed by using standard methods. The primarily used antibodies included anti-E-cadherin (610181), anti-Vimentin (550513), and anti-N-cadherin (610921) from BD Pharmingen (San Diego, CA, USA); anti-Histone H3 (#14269), anti-snail (#3879), anti-β-catenin (#9582), and anti-GAPDH (2118S) from Cell Signaling Technology (Danvers, MA, USA); anti-MOSPD2 (NBP1-91438) from Novus Biologicals (Novus, CO, USA); anti-β-actin (#A5441) from Sigma-Aldrich (Sigma-Aldrich, MA, USA).

### Wound-healing assays

NPC cells were seeded into 6-well plates at a density of 5 × 10^5^ cells/well and allowed to grow to 80–90% confluent in complete medium at 37 °C. Then all cells 16 h starvation in a serum-free medium. The monolayers were disrupted by scraping them with a sterile 10-μl tip. The cells’ migration progress into the wound was photographed by an inverted microscope (Leica DMI4000B, Wetzlar, Germany) at 0, 24, and 48 h. Each dish was measured three times at each wound.

### Migration and invasion assays

A total of 1 × 10^5^ (for C666-1, SUNE-1) or 4 × 10^4^ (for CNE-1 and CNE-2) cells in 200 μl of serum-free RPIM 1640 were transferred to the superstratum chamber inserts with 8 μm pore size fibronectin-coated polycarbonate membrane (Corning, Corning, NY, USA) and 750 μl of RPIM 1640 containing 20% FBS was added into the chamber below. CNE-1, CNE-2, SUNE-1 cells were incubated for 18 h, respectively, while C666-1 cells were incubated for 24 h. Then the upper chamber soaks in 4% PFA Fix Solution (JingXin, Guangzhou, China) for 15 min with removing the cells on the higher surface of the membrane, after the sample was dried stained with crystal violet for 10 min, and then the number of cells on the membrane was counted by inverted microscope (×100 magnification). Cell invasion assays were performed as the migration assay, except the upper membrane was coated with Matrigel before the step seeds cell 3 h (BD Biosciences, Franklin Lakes, NJ, USA).

### Tissue microarray (TMA) and in situ hybridization (ISH)

Human tissue specimens microarrays were purchased from Shanghai Outdo Biotech CO, LTD. NPC specimens and normal-looking squamous mucosa specimens were used to quantify EBV-miR-BART-22 expression. Our study was approved by the Shanghai Outdo Biotech Company ethics committee.

ISH was utilized to determine the relative expression of EBV-miR-BART-22 in 132 following specimens following the manufacturer’s instructions. In brief, the TMAs were disposed of in the process of dewaxing, rehydration, and digestion, followed by hybridizing with the specific EBV-miR-BART-22 probe by miRCURY LNA microRNA Detection probes (Exiqon, Vedbaek, Denmark). The samples were incubated with anti-Digoxin-AP (Roche, Basel, Switzerland) and stained with NBT/BCIP (Roche, Basel, Switzerland). Finally, the expression of EBV-miR-BART-22 was quantified and imaged in TMAs from NPC.

### Immunohistochemistry (IHC)

The paraffin-embedded tissues were cut into 4-μm thickness sections, and the procedures of immunohistochemical staining of E-cadherin, N-cadherin, Vimentin, β-catenin, MOSPD2. After being baked at 65 °C for 1–2 h, dewaxed in dimethyl benzene, and hydration in a gradient of alcohol concentrations, the tissues were subjected to citrate antigen recovery buffer 125 °C for 10 min. Endogenous peroxidase activity and nonspecific binding of antibodies were blocked by 3% H_2_O_2_ and 1% goat serum albumin, respectively. The samples were then probed with primary antibodies at 4 °C for 18 h. The slides were incubated with appropriate secondary antibodies to bind the primary antibodies, followed by an avidin-biotin technique with diaminobenzidine (DAB) visualization and counterstaining with hematoxylin. The immunostained tissue microarray slides were photographed by the Leica SCN400 slide scanner (Meyer Instruments, Houston, TX, USA).

### Immunofluorescence (IF)

The cells were cultured on coverslips for 12 h, washed with cold PBS three times, and fixed in 4% PFA Fix Solution for 10 min. Subsequently, the cells were blocked with goat serum for 1 h at room temperature and incubated with the anti-β-catenin, E-cadherin, and Vimentin antibody in antibody diluents (Dako) at 4 °C overnight. After being washed, the samples were incubated with the Alex Fluor 488-Donkey anti-rat IgG (H + L; 1:150, Life Technologies, #A21208) and Alex Fluor 594-Donkey anti-rabbit IgG (1:150, Life Technologies, #R37119) for 1 h in the dark at room temperature. The samples were stained with DAPI (4′,6-diamidino-2-phenylindole, Sigma-Aldrich, MO, USA) for 8 min to marker the nucleus and the slices were digitally photographed with a confocal microscope (Leica TCS SP8, Wetzlar, Germany).

### Bioinformatic prediction of microRNA targets

The candidate targets of BART22 were initially predicted by Targetscan Custom (http://www.targetscan.org/vert_50/seedmatch.html) and Vir-Mir (http://alk.ibms.sinica.edu.tw/cgi-bin/miRNA/miRNA.cgi). The 3′UTR of the potential target genes predicted by both were obtained from NCBI and subjected to DIANA (http://www.microrna.gr/microT).

### In vivo metastasis assay

We chose the hepatic metastases models by splenic injection to evaluate tumor metastasis. BALB/c-nu female mice (4–6 weeks, 16–18 g) were housed in a specific pathogen-free environment. The NPC cells (5 × 10^5^ in 25 μl of 33% Matrigel) were injected into the spleens of nude mice using insulin syringes (NIPRO, Bridgewater, NJ, USA) as previously reported. After 30 days of feeding and then sacrificed. The liver and spleen of each mouse were weighed, and the metastatic nodules in each liver were counted.

### Lentiviral construction and transduction

Lentiviral particles (GV and U6-MCS-SV40-EGFP-IRES-puromycin) containing EBV-miR-BART-22 precursors and lentiviral particles (GV and hU6-MCS-UbiquitinEGFP-IRES-puromycin) containing a reverse complement of EBV-miR-BART-22 and their control vectors were constructed by Shanghai Genechem Co., Ltd. (Shanghai, China). CNE-1, CNE-2, and SUNE-1 cells were transfected with a recombinant lentiviral vector to upregulate EBV-miR-BART-22 expression, and C666–1 cells were transfected with a lentiviral vector to downregulate EBV-miR-BART-22 expression. The transfection efficiency was checked using qPCR assay. For the rescue assay, CNE-1-BART-22 cells, CNE-2-BART-22 cells, and SUNE-1-BART-22 cells were transfected with the β-catenin lentiviral vector siQ0001 (Guangzhou RiboBio Co., Ltd., Guangzhou, China) or normal control. The NPC cells were transduced with lentiviruses. After 7 days, 1–2 μg/ml puromycin (Sigma-Aldrich, St Louis, MO, USA) was added to the culture and lasted for 12 days. qRT-PCR was performed to analyze the EBV-miR-BART-22 expression.

### Luciferase reporter assay

The MiTarget microRNA 3′-UTR target vector (pEZX-MT01) containing full-length 3′-UTR of MOSPD2 with the binding site for EBV-miR-BART-22 (wild-type 3′-UTR) was produced by GeneCopoeia (GeneCopoeia, Rockville, USA). For luciferase reporter assays, wt or mut 3′-UTR vector was co-transfected with EBV-miR-BART-22 mimic or nonspecific mimic control (GeneCopoeia, Rockville, USA) into HEK 293T cells, respectively. Luciferase activity was measured 48 h after transfection using Luc-Pair miR Luciferase Assay Kit (GeneCopoeia, Rockville, USA) on Panomics Luminometer.

### Statistical analysis

All the assays and experiments were performed at least thrice with triplicate repeats. All results were expressed as the mean ± standard deviation (S.D.). One-way analysis of variance (ANOVA) or Student’s test was used to perform statistical differences. All statistical analyses were performed using SPSS 24 (Chicago, IL, USA) or GraphPad Prism 8.0 (GraphPad Software, La Jolla, CA, USA).

## Results

### EBV-miR-BART-22 is upregulated in NPC tissues and EBV-positive cell lines

**Fig. 1:**
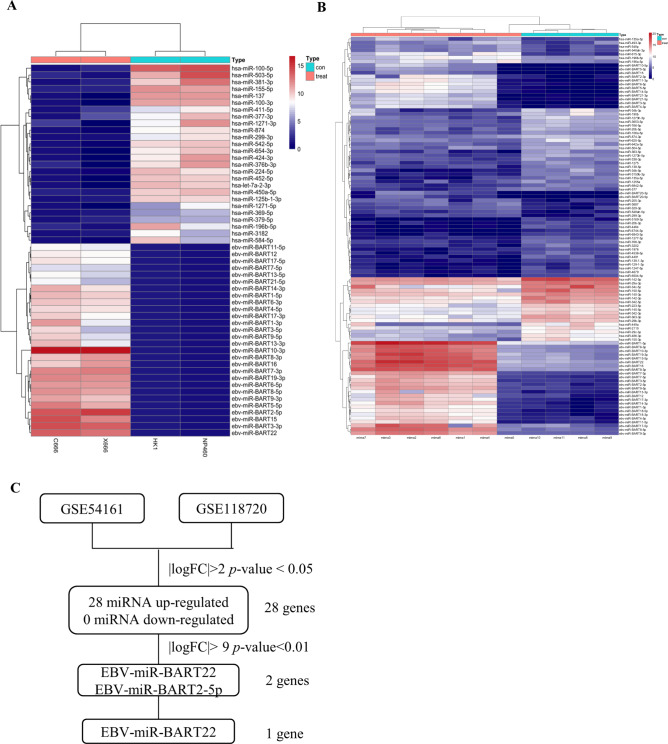
EBV-miR-BART22 expression is upregulated in nasopharyngeal carcinoma. **A** The cluster heat maps showing the GSE54161 different expressed microRNAs in 2 lines of EBV positive and 2 lines of EBV-negative nasopharyngeal carcinoma cells. The blue and red stripes represent downregulated and upregulated microRNAs, respectively. **B** The cluster heat maps showing the GSE118720 different expressed microRNAs in 7 NPC biopsy specimens and 4 normal nasopharyngeal mucosal specimens. The blue and red stripes represent downregulated and upregulated microRNAs, respectively. **C** Microarray analysis was performed to identify EBV and nasopharyngeal carcinoma-associated microRNAs. Expression levels were analyzed in GSE54161, GSE118720. To narrow the range of values, the cut off with *p*-value < 0.01 and |fold change (FC)| > 2, as shown in the flowchart. These data were integrated with tighter standards to identify the potential esophageal carcinoma and EBV-associated microRNAs (two microRNAs).

To identify potential EBV-associated prognostic miRNAs in NPC, we first analyzed miRNA datasets (ID: GSE54161) from the Gene Expression Omnibus (GEO) public database. Comparison of EBV-associated miRNAs that underwent expression changes from EBV-negative to EBV-positive cells was conducted by the R software package pheatmap (v1.0.12) (Fig. 1A). Then we analyzed the miRNA datasets (ID: GSE118720) to identify differentially expressed miRNAs in 7 NPC biopsy specimens and 4 normal nasopharyngeal mucosal specimens (Fig. 1B). The miRNAs were considered significantly differentially expressed when setting the transcript expression change threshold to *P* value < 0.05 and |log_2_(fold change)| > 2 cut-offs at least. To further filter miRNAs based on significance level in GSE54161 and GSE118720, we narrowed the threshold values to *P* value < 0.01 and |log_2_(fold change)| > 9 cut-offs, as shown in the flowchart. Then we obtained 2 miRNAs (EBV-miR-BART22 and EBV-miR-BART2-5P). Interestingly, little has been reported on the effects of EBV-miR-BART-22 in NPC (Fig. 1C). These results suggested that high expression of EBV-miR-BART-22 may play an essential role in EBV-associated NPC progression and act as a novel prognostic factor for NPC patients.

### A high level of EBV-miR-BART-22 is associated with an unfavorable prognosis in NPC patients

**Fig. 2:**
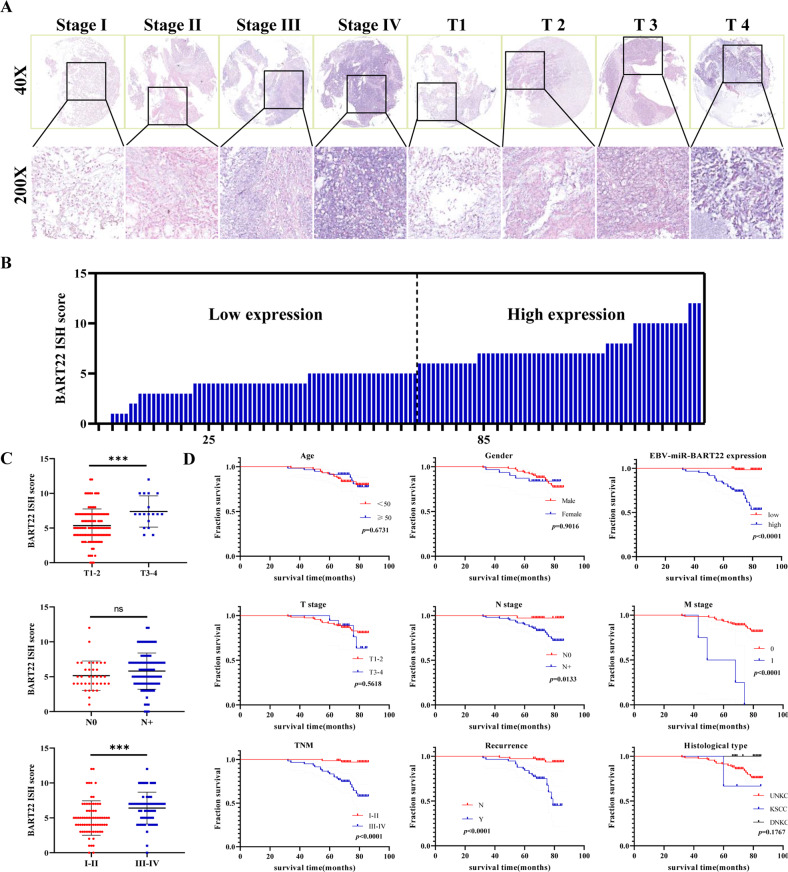
EBV-miR-BART22 is abnormally expressed in advanced NPC patients and related to clinical indicators. **A** Levels of EBV-miRNA-BART22 expression in NPC tissue chips are shown under both low and high magnifications. **B** The ISH score was calculated for each NPC sample. Tumors with an expression level lower than 6 points are considered low expression, while 6 points or more are considered high expression. **C** Graph showing the ISH staining scores and pathological clinical stage in 132 NPC patients. **D** Kaplan–Meier curve analysis revealed that NPC patients with high EBV-miRNA-BART22 expression had a shorter survival time than NPC patients with low EBV-miRNA-BART-22. The survival time was significantly associated with N stage, M stage, TNM stage, Recurrence, but not with age, gender, T stage, Histological type.

To further confirm the prognostic value of the EBV-miR-BART-22 signature in addition to its clinical value in NPC, ISH experiments were carried out in a tissue chip containing 132 NPC tissues to probe the expression of EBV-miR-BART-22 (Fig. 2A, B). In addition, we explored the potential relationship between the clinical features of NPC patients and EBV-miR-BART-22 expression. There may be a correlation between the expression of EBV-miR-BART-22 and clinical stage (*P* < 0.001), T stage (*P* = 0.005), M stage (*P* = 0.031), recurrence (*P* < 0.0001), and histological type (*P* = 0.027). Nevertheless, the other clinical indicators, including age, gender, and N stage, did not correlate with EBV-miR-BART-22 expression (Table S1). An apparent positive correlation was found between the expression of EBV-miR-BART-22 and T stage (*P* < 0.0009) and TNM stage (*P* = 0.0008) (Fig. 2C). Kaplan–Meier survival curves indicated that patients with low expression of EBV-miR-BART-22 (*P* < 0.0001), N0 stage (*P* = 0.0133), M0 stage (*P* < 0.0001), TNM stages I/II (*P* < 0.0001), and no recurrence (*P* < 0.0001) had longer survival times than the remaining patients (those with clear overexpression of EBV-miR-BART-22, N + stage, M1 stage, TNM stages III/IV, and recurrence, respectively) (Fig. 2D). Multivariate Cox regression analyses revealed that high EBV-miR-BART-22 expression was an independent predictor and was significantly associated with poor NPC patient outcomes (HR = 0.117, *P* = 0.045) (Table S2). These results suggest that EBV-miR-BART-22 could contribute to the development of NPC tumorigenesis, and that overexpression of EBV-miR-BART-22 is significantly associated with a poor prognosis in NPC patients.

**Fig. 3:**
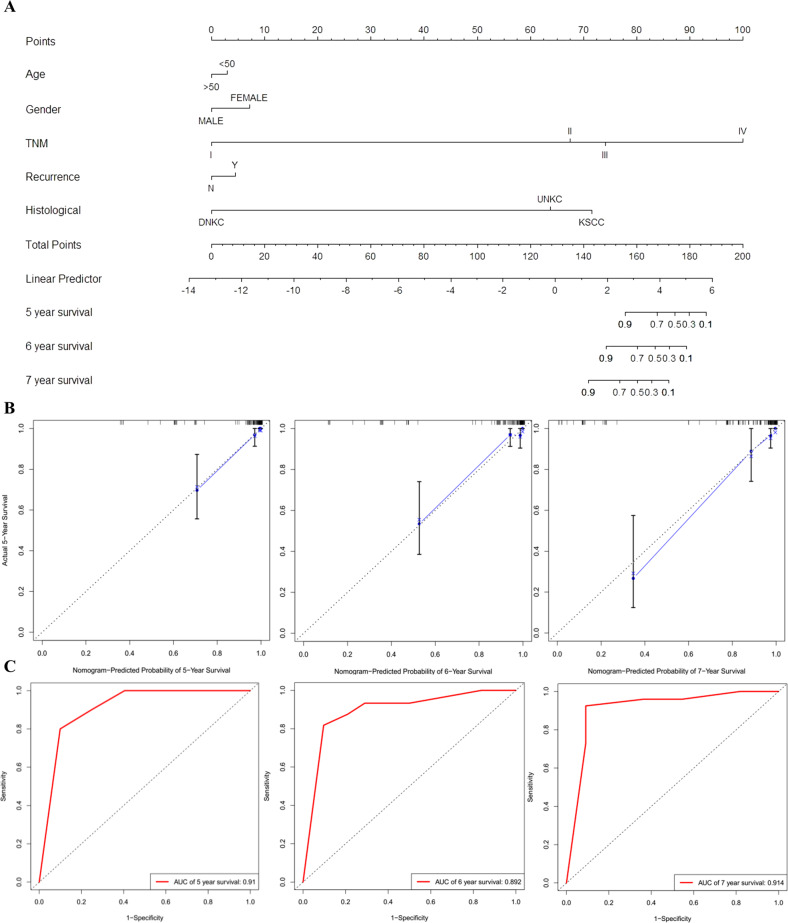
A nomogram prediction model was constructed based on the expression of BART22 in NPC. **A** The nasopharyngeal carcinoma survival nomogram. (To use the nomogram, an individual patient’s value is located on each variable axis, and a line is drawn upward to determine the number of points received for each variable value. The sum of these numbers is located on the Total Points axis, and a line is drawn downward to the survival axes to determine the likelihood of 5-, 6- or 7-year survival). Risk of survival time can be determined by assigning points for each variable by drawing a line upward from the corresponding variable to the points line, summing the points, and identifying the prediction of survival time associated with the total points line. EBV-miR-BART-22, the level of tissue EBV-miR-BART-22 before treatment. **B** Calibration plots for predicting for NPC OS at 5-, 6-, and 7-year. The blue dotted line indicates the ideal nomogram; blue X indicates the bootstrap-corrected estimates; vertical bars indicate the 95% CIs. **C** ROC curves of the 5-, 6-, and 7-year nomograms of NPC patients. The red bars represent new nomogram-predicted OS, whereas the black bars represent TNM stage predicted OS.

To provide clinicians with a quantitative method to predict the OS time of NPC patients with low or high EBV-miR-BART-22 expression, we constructed a model that integrated the EBV-miR-BART-22-based classifier and clinicopathological risk factors. According to the multivariate Cox risk regression analysis, we created a predictive nomogram model including age, gender, TNM stage, EBV-miR-BART-22, recurrence, and histological type for predicting the 5-, 6-, and 7-year OS rates of the individual NPC patients (Fig. 3A). The calibration plots of the nomogram indicated a good concordance of the nomogram-predicted and actual 5-, 6-, and 7-year OS values (Fig. 3B). We further performed ROC curve analysis for each screen, which yielded 5-, 6-, and 7-year AUC values of 0.91, 0.892, and 0.914, respectively (Fig. 3C). These results demonstrate that the EBV-miR-BART-22 assessment combined with TNM staging could constitute a risk-scoring system to predict NPC patient prognosis.

### EBV-miR-BART22 induces EMT to promote NPC cell metastasis in vitro and in vivo

**Fig. 4:**
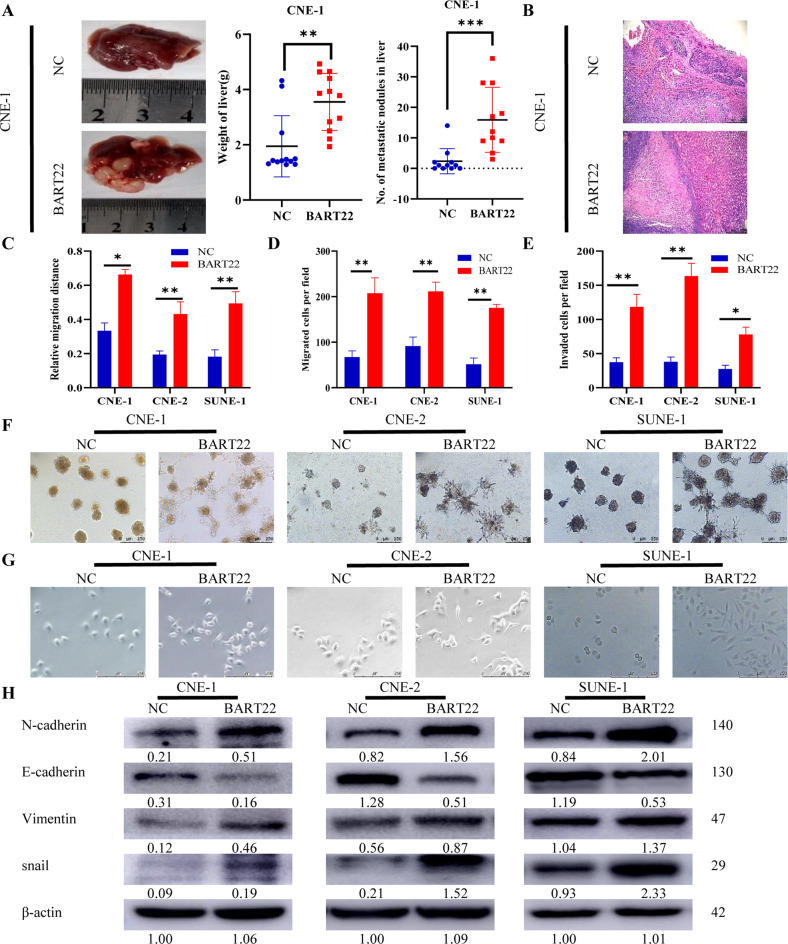
Overexpression of EBV-miR-BART22 promotes the migration, invasion, and EMT of NPC cells. **A** A representative bright-field image of the livers was shown. Liver weights and metastatic modules number in nude mice that received transplants of indicated cells. Bars correspond to mean ± standard deviation (SD), **P < 0.01. ***P < 0.001. (**B**). Representative H&E staining of indicated spleen orthotopic tumors is shown. **C** CNE-1, CNE-2, and SUNE-1 cells and appropriate controls were used in a wound-healing assay. **D** Quantification of indicated migrating cells in 5 random fields analyzed by Transwell assays, respectively. **E** Quantification of indicated invasion cells in 5 random fields analyzed by invasion assays, respectively. **F** Representative micrographs of indicated cells grown on matrigel for 10 days in 3D spheroid invasion assay. **G** Cells morphology was evaluated by phase-contrast microscopy. **H** Expression of epithelial cell marker and mesenchymal cell marker in indicated cells were examined by WB analysis. β-actin was used as a loading control.

Lentivirus transfection was utilized to construct NPC cell lines stably overexpressing EBV-miR-BART-22 EBV (−) (CNE-1, CNE-2, SUNE-1). The relative transcript abundance of EBV-miR-BART-22 in NPC cells was determined using qPCR (Fig. S1A). To further determine the role of EBV-miR-BART-22 in metastasis in *vivo*, mouse spontaneous spleen-liver metastasis models were established. Furthermore, mice injected with EBV-miR-BART-22-overexpressing cells were more likely to exhibit metastasis. Liver metastasis was dramatically promoted, and the weight of the liver was increased with EBV-miR-BART-22 overexpression (Figs. 4A and S2B). Subsequently, the spleen and liver were fixed for H&E staining to identify small metastatic lesions under a microscope. Approximately half the mice injected with EBV-miR-BART-22-expressing NPC cells developed such metastatic colonies that could be viewed under a microscope (Fig. 4B). All these results confirmed that EBV-miR-BART-22 could promote cell metastasis in vivo. In wound-healing assays and transwell migration assays, overexpression of EBV-miR-BART-22 observably promoted cell migration in EBV (−) NPC cell lines (Figs. 4C, D and S2A, B). Moreover, the invasion assay of EBV (−) NPC cell lines revealed that EBV-miR-BART-22 promotes NPC cell invasion (Figs. 4E and S2B). Moreover, in 3D culture, EBV-miR-BART-22 overexpressing cells displayed stronger invasiveness (Fig. 4F).

To further investigate the impact of EBV-miR-BART-22 on EMT in NPC cells, we overexpressed EBV-miR-BART-22 in CNE-1, CNE-2, and SUNE-1 cells. The NPC cells overexpressing EBV-miR-BART-22 displayed a mesenchymal-like morphology (spindle-like and fibroblastic phenotype), while control vector-transfected NPC cells showed an epithelial-like morphology (cobblestone-like) (Fig. 4G). We investigated the expression of E-cadherin, N-cadherin, and vimentin, as well as the snail, a characteristic marker of EMT. Western blotting demonstrated that the upregulation of EBV-miR-BART-22 markedly decreased the expression of E-cadherin but increased the expression of N-cadherin and vimentin in CNE-1-BART22, CNE-2-BART22, and SUNE-1-BART-22 cells compared with the relative control cells (Fig. 4H). We then performed IF staining for EMT markers (E-cadherin and vimentin). Fluorescence microscopy examinations showed that the upregulation of EBV-miR-BART-22 modestly increased the expression density of EMT markers in CNE-1-BART-22, CNE-2-BART22, and SUNE-1-BART22 cells as compared with that of the corresponding mock control cells (Fig. S4). Western blotting was used to detect the expression of EMT-related proteins in animal tumor tissues. The results were consistent with the in vitro assays (Fig. S5A). IHC staining of tumor sections of xenografts and metastasized organs demonstrated that the upregulation of EBV-miR-BART-22 overtly reduced E-cadherin expression but increased the expression of N-cadherin and vimentin in CNE-1-BART22 tumors compared with the CNE1-mock control tumors (Fig. S5B). These results confirmed that EBV-miR-BART-22 could promote NPC cell migration and invasion through the induction of EMT.

### Knockdown of EBV-miR-BART22 expression induces restoration of the epithelial phenotype

**Fig. 5:**
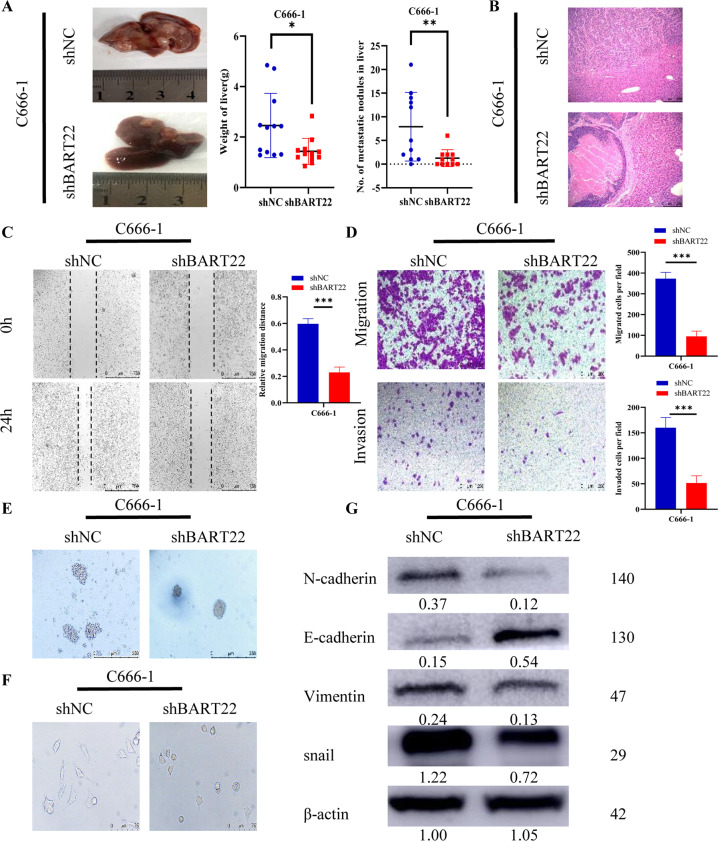
Overexpression of EBV-miR-BART22 promotes the migration, invasion, and EMT of NPC cells. **A** The representative image of the distant liver tumor was displayed. *P < 0.05. **P < 0.01. **B** H&E staining of the spleen tumors (magnification, ×100, Scale bar, 100 μm) were showed. **C** Representative photomicrographs of scratch wounds at 0 and 24 h after wounds were made. Scale bar = 100 mm (*n* = 3 each). Data are means ± S.E.M. *P < 0.05, **P < 0.01. **D** The C666-1 cellular migration and invasion capability were assessed by transwell migration and Boyden chamber invasion assays (**E**). Representative micrographs of indicated cells grown on matrigel for 10 days in 3D spheroid invasion assay. **F** Cells morphology was evaluated by phase-contrast microscopy. **G** The expression of epithelial and mesenchymal cell markers was examined by WB analysis in EBV-miR-BART-22 inhibition C666-1 and the corresponding control cells.

We then stably suppressed EBV-miR-BART-22 in EBV ( + ) NPC cell lines (C666-1) by lentiviral transfection (Fig. S3A). EBV-miR-BART-22 knockdown cells displayed metastasis and decreased liver weight (Figs. 5A and S3B). Primary tumor derived from C666-1-shNC cells displayed a more invasive phenotype than those derived from C666-1-shBART22 cells (Fig. 5B). Upon transfection with shRNA EBV-miR-BART-22, cell migration and invasion were evaluated by wound-healing assay, transwell assay, and invasion assay at the same time. The experiments strongly suggested that EBV-miR-BART-22 knockdown significantly decreased the motility of C666-1-shBART22 cells compared to the control (Fig. 5C, D). In 3D culture, C666-1-shNC cells displayed stronger invasiveness than C666-1-shBART22 cells (Fig. 5E). C666-1-shBART22 cells were transformed from spindle-like and fibroblastic to a cobblestone-like phenotype after inhibiting EBV-miR-BART-22 (Fig. 5F). Western blotting demonstrated that the downregulation of EBV-miR-BART22 in C666-1 cells increased E-cadherin expression but decreased the expression of N-cadherin and vimentin compared with the control (Fig. 5G). IF and IHC staining were also performed to detect the expression of EMT-related proteins in vivo and in vitro assays (Figs. S4 and S5B). Altogether, the results suggest that EBV-miR-BART22 promotes cell motility and invasiveness by driving EMT in NPC cells.

### EBV-miR-BART22 mediates EMT to promote cell migration via the Wnt/β-catenin signaling pathway

**Fig. 6:**
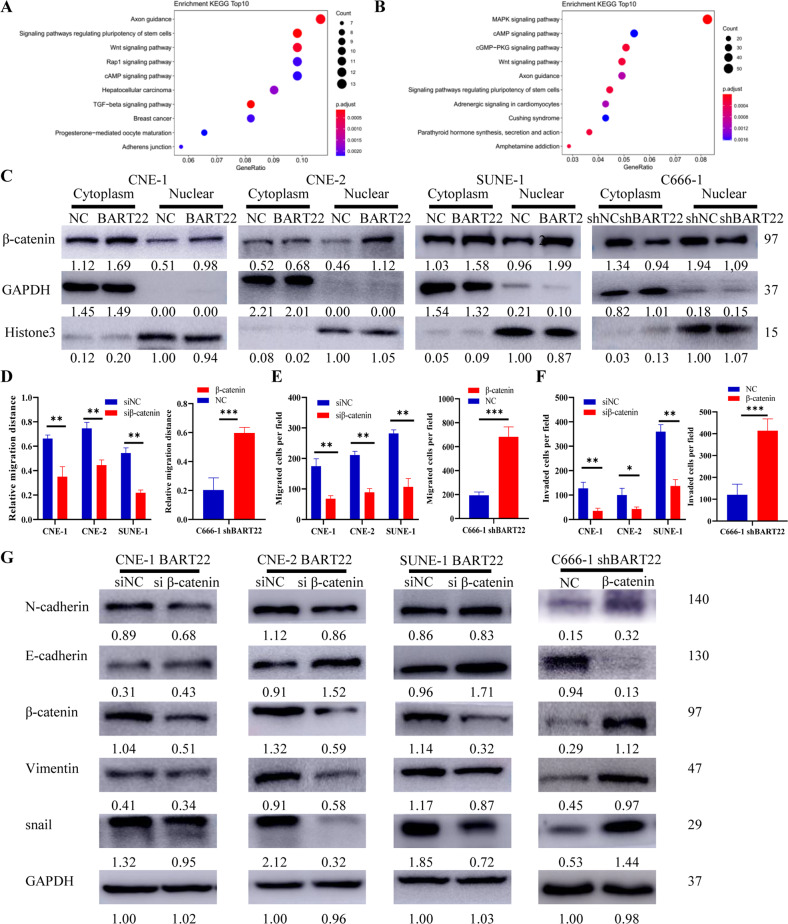
EBV-miR-BART22 induces β-catenin abnormally activated in the NPC cell lines. **A** Pathway based on the KEGG and TargetScan databases. **B** Pathway based on the KEGG and DIANA databases. **C** The nuclear fraction of indicated cells was analyzed by WB analysis. **D**–**F** Cells were treated with β-catenin siRNA and plasmid β-catenin for 72 h, and then performed wound-healing assays and Transwell/migration assay. *P < 0.05. **P < 0.01. **G** Expression of epithelial cell marker, mesenchymal cell marker, and β-catenin in indicated cells were examined by WB analysis. GAPDH was used as a loading control.

To identify other signaling pathways potentially regulating the expression of the miRNA, we next subjected the different datasets to KEGG pathway enrichment analysis, and the results indicated that the Wnt signaling pathway plays a key role in tumorigenesis in EBV-miR-BART22-related carcinoma (Fig. 6A, B). According to a previous study, the Wnt/β-catenin signaling pathway, also called the canonical Wnt signaling pathway, is a conserved signaling axis participating in diverse physiological processes such as proliferation, differentiation, apoptosis, migration, invasion, and tissue homeostasis [29]. Therefore, we sought to examine the potential role of the Wnt/β-catenin pathway in EBV-miR-BART-22-induced EMT in NPC cells. We measured the impact of EBV-miR-BART-22 on the nuclear translocation of β-catenin. The results showed that EBV-miR-BART-22 could increase the elevated nuclear β-catenin level (Fig. 6C). There was a global increase of β-catenin expression, we calculated the ratio of nuclear/cytoplasmic β-catenin, and β-catenin increased more in the nucleus than in the cytoplasm. β-Catenin increases expression is induced more strongly in the nucleus. The ratio of nuclear/cytoplasmic β-catenin was increased in the different EBV-miR-BART-22 overexpressing cells. To validate our experimental results, we used siRNA interference to decrease β-catenin expression in CNE-1-BART22, CNE-2-BART22, and SUNE-1-BART22 cells (Fig. S6A, B) and used the plasmids to promote β-catenin expression in C666-1-shBART22 cells (Fig. S6C, D). The results showed that interference with β-catenin expression impaired the EBV-miR-BART-22-induced migration and invasion of CNE-1, CNE-2, and SUNE-1 cells (Figs. 6D–F and S7A, B). β-Catenin depletion also interfered with EBV-miR-BART22-induced EMT according to the Western blotting results (Fig. 6G). These results indicate that EBV-miR-BART22 promotes NPC metastasis by activating the Wnt/β-catenin signaling pathway.

### EBV-miR-BART22 targets MOSPD2 directly in vitro and in vivo

**Fig. 7:**
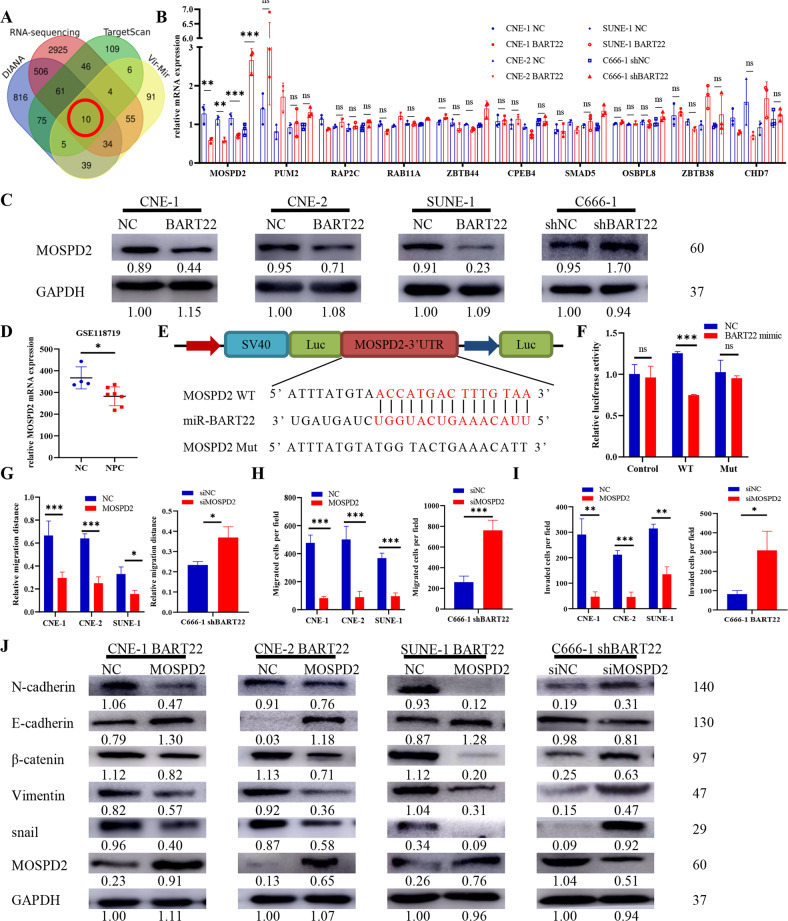
MOSPD2 is a direct target of EBV-miR-BART22. **A** A Venn diagram depicting the overlap of target genes predicted by TargetScan (green), RNA-sequencing (red), DIANA (blue), and Vir-Mir (yellow). Putative target genes identified by this approach are listed. **B** Quantitative reverse transcription-polymerase chain reaction (qRT-PCR) analysis of EBV-miR-BART22 regulation on target genes in nasopharyngeal carcinoma cells. **C** Expression of MOSPD2 in indicated cells was examined by WB analysis. **D** The relative expression levels of MOSPD2 in normal nasopharyngeal tissues and nasopharyngeal tumors in the GEO data set for nasopharyngeal carcinoma. **E** The construction of luciferase reporter vectors with inserted WT or mutant MOSPD2 3′-UTR sequences. **F** Changes in the normalized reporter gene activities of WT and mutant MOSPD2 3′-UTR luciferase reporter constructs responded to the co-transfection of negative control (NC) or EBV-miR-BART22 mimic in T293 cells. **G** Representative photomicrographs of scratch wounds at 0 and 24 h after wounds were made. Quantitative measurement of wound gaps by Photoshop software. **H** Quantification of indicated migrating cells in 5 random fields analyzed by Transwell assays, respectively. **I** Quantification of indicated migrating cells in 5 random fields analyzed by invasion assays, respectively. **J** Expression of epithelial cell marker, mesenchymal cell marker, β-catenin, and MOSPD2 in indicated cells were examined by WB analysis. GAPDH was used as a loading control.

An online bioinformatic predictor and literature retrieval were used to identify the potential targets of EBV-miR-BART-22. DIANA, TargetScan Custom, Vir-Mir, and RNA sequencing were used to predict downstream targets of EBV-miR-BART-22, and 10 common genes were identified (Fig. 7A). Of these, only the expression of MOSPD2 was significantly decreased in EBV-negative NPC cells with ectopic expression of EBV-miR-BART-22 versus control cells, whereas it was upregulated in EBV-positive NPC cells with genetic downregulation of EBV-miR-BART-22 (Fig. 7B). Western blot analysis was conducted and confirmed that compared with the negative control, EBV-miR-BART-22 decreased the expression of MOSPD2 (Fig. 7C). The GEO arrays (GSE118719) also showed low expression of MOSPD2 in carcinoma tissues (Fig. 7D). The dual-luciferase reporter assay revealed that co-transfection of a dual-luciferase reporter containing wild-type MOSPD2 and EBV-miR-BART-22 mimic significantly decreased luciferase activity. We further assessed these MOSPD2 binding sites by introducing mutations into the binding sites predicted by bioinformatic analysis (Fig. 7E, F). The IHC results showed that the expression of MOSPD2 was reduced in metastatic tumors formed by EBV-miR-BART-22 compared with metastatic tumors formed by control cells (Fig. S8). Altogether, these data indicate that MOSPD2 is a direct cellular target of EBV-miR-BART-22 in NPC.

### MOSPD2 mediates EBV-miR-BART-22-induced NPC cell migration, invasion, EMT, and Wnt/β-catenin signaling

To explore the molecular mechanism of MOSPD2 in regulating NPC cell metastasis, we used plasmids to promote MOSPD2 expression in CNE-1-BART22, CNE-2-BART22, and SUNE-1-BART22 cells (Fig. S9A) and siRNA interference to decrease MOSPD2 expression in C666-1-shBART22 cells (Figs. S9B and S9C). MOSPD2 overexpression reduced the migration and invasion capacity of CNE-1-BART22, CNE-2-BART22, and SUNE-1-BART22 cells (Figs. 7G–I and S9D, E). MOSPD2 overexpression also increased the expression of an epithelial marker (E-cadherin) and decreased the expression of mesenchymal markers (vimentin, N-cadherin) and β-catenin (Fig. 7J). These findings indicated that MOSPD2 is the functional target of EBV-miR-BART22 and that MOSPD2 overexpression significantly reverses EBV-miR-BART-22-induced migration, metastasis, and EMT in vitro.

**Fig. 8:**
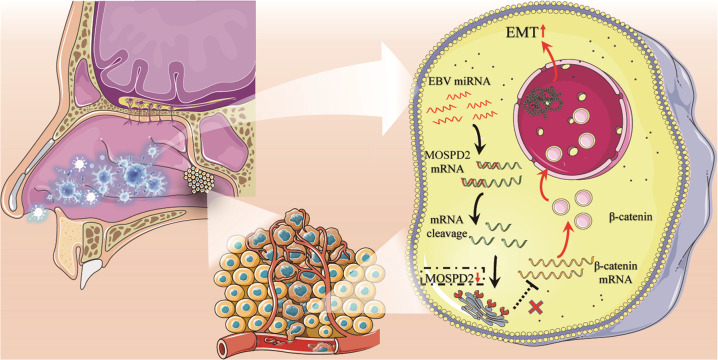
The schematic summary demonstrating the role of EBV-miR-BART22 in NPC metastasis. EBV-miR-BART-22 plays an oncogenic role by driving the EMT and metastasis of NPC by targeting MOSPD2 and activating the Wnt/β-catenin pathway.

Altogether, our study found that the expression of EBV-miR-BART-22 in NPC is significantly increased, and that EBV-miR-BART-22 expression is negatively correlated with the clinical prognosis of NPC. EBV-miR-BART-22 can promote the metastasis, invasion, and EMT of NPC cells by targeting MOSPD2 to activate the Wnt/β-catenin signaling pathway (Fig. 8). Thus, EBV-miR-BART-22 is a potential prognostic biomarker and new antimetastatic target for NPC.

## Discussion

NPC is an EBV infection-related malignancy with unique geographic distribution. Most research focuses on EBV-associated proteins, such as EBNA1, LMP1, LMP2A, and LMP2B. However, it may be related to other essential factors, such as EBV-encoded microRNA (EBV-miRNA). Some BART microRNAs with rich abundance in NPC have generated additional interest in investigators who contribute to a better understanding of microRNA functions. Prior studies have reported that the high expression of EBV-miR-BART-20-5p in NPC tissues is positively correlated with a poorer 5-year recurrence-free survival rate [30]. The high expression of EBV-miR-BART8 and EBV-miR-BART-20-5p can enhance the invasiveness of nasal NK/T-cell lymphoma [31]. NPC patients with higher EBV-miR-BART7-3p expression had an increased risk of recurrence and worse outcomes in nasopharyngeal carcinoma [32]. In addition, nanoparticles targeting EBV-miR-BART7-3p could constitute a new strategy for treating NPC in mouse models [28]. Plasma BART8-3p is also a meaningful biomarker for predicting the risk of recurrence and metastasis in NPC patients [33]. A number of studies have already reported that EBV-miR-BART-2-5p is correlated with NPC [34, 35]. With comprehensive bioinformatics analysis and meta-analysis results, we selected EBV-miR-BART-22. Here, we measured the expression level of EBV-miR-BART-22 on 132 clinical samples from patients with NPC (Fig. 2A). We found that high expression of EBV-miR-BART-22 was significantly correlated with distant metastasis and a poor prognosis (Fig. 2C, D). It is abundant in most early NPC patients, and it has been found to be increased even one year before clinical diagnosis, indicating that EBV-miR-BART22 has an essential biological role in the development and progression of NPC [21, 36]. The high-level expression of EBV-miR-BART-22 is closely related to the recurrence and metastasis of NPC, and EBV-miR-BART-22 is also an independent predictor of a poor prognosis in NPC (Tables S1 and S2). Thus, our findings shed new light on the association of EBV BART miRNAs with NPC metastasis.

In this study, Kaplan–Meier analysis demonstrated that NPC patients with high EBV-miR-BART-22 expression showed poor clinical outcomes. Although EBV-miR-BART-22 expression has been shown to be associated with poorer overall survival rates in a previous study [37], we revealed for the first time that EBV-miR-BART-22 can be used to predict the survival time of NPC patients. Our multivariate analysis also revealed that high EBV-miR-BART-22 expression was independent factor predicting an unfavorable prognosis in NPC patients. Thus, we developed a comprehensive predictive model incorporating age, gender, TNM stage, EBV-miR-BART-22 expression, recurrence, and histological type clinical parameters to predict the OS probability of individual NPC patients (Fig. 3A). Calibration plots indicate that our model is highly specific, stable, accurate, and amenable for use in the clinical setting to facilitate decision-making. Moreover, our clinical follow-up data began in 2010-2011 and ended in 2017, and most patients survived longer than 5 years, so our predictive model is fit to predict 5-, 6-, and 7-year OS rates in NPC patients. Currently, the prediction of NPC prognosis is mainly based on clinical TNM staging. However, NPC patients with the same clinical stage often present different clinical outcomes, suggesting that the TNM stage is insufficient to precisely predict the prognosis of this disease [38, 39]. Compared to the conventional TNM staging system, the nomogram incorporating EBV-miR-BART-22 could better predict OS (Fig. S10A, B). Our clinical data demonstrate the critical clinical significance of EBV-miR-BART-22 in NPC metastasis. To the best of our knowledge, this is the first study reporting EBV-miR-BART-22 as a predictor for the prognosis of NPC.

EMT is the fundamental biological process that transforms polarized epithelial cells into interstitial cells in a dynamic way, including actin cytoskeleton remodeling and enhancement of the cell metastasis capability [40]. It is considered to be the first step in tumor metastasis. Previous studies have confirmed that the Notch, PI3K/Akt, MAPK/ERK, and Wnt pathways regulate EMT and promote cell migration [41]. In the present research, we demonstrated that EBV-miR-BART-22 in NPC cells significantly reduced the expression of E-cadherin, an important epithelial phenotype marker, and increased the expression of the mesenchymal phenotype markers N-cadherin and vimentin (Figs. 4G and 5G). In clinical tumor samples, it has been reported that the expression of E-cadherin is downregulated and is negatively correlated with lymph node metastasis of NPC [42]. However, because cells undergoing EMT may share some molecular and morphological markers with the surrounding stromal cells [43], EBV-miR-BART-22 might affect motility through other routes and control EMT in turn. Overall, our findings indicate that EBV-miR-BART-22 promotes EMT and increases NPC cell motility and invasiveness.

Abnormalities of the Wnt/β­catenin pathway occur extensively in many types of malignancies [44]. Key inactivated transcriptional regulators of negative Wnt/β­catenin signaling lead to abnormally sustained activation of the Wnt/β­catenin signaling pathway in NPC metastasis [45]. Therefore, it is important to identify more silenced negative regulators of the Wnt/β­catenin signaling pathway, which might help provide clinicians with beneficial NPC treatment decisions. The canonical Wnt signaling pathway regulates the transcription of many genes, and β-catenin is the central mediator of this pathway. The Wnt/β-catenin signaling pathway can signal β-catenin to accumulate and enter the nucleus and activate downstream TCF/LEF family regulatory factors to play a corresponding regulatory role [46]. According to a recent study, EBV-miR-BART-10-3p and EBV-miR-BART-22 can activate Wnt signaling by reducing the expression of β-TrCP to induce the migration and metastasis of gastric carcinoma cells [47]. In our research, we found that APC and DKK1were differentially expressed in EBV-associated GC but not in the EBV-positive and EBV-negative NPC cell lines, and there are different EBV-miR-BART22 regulation models in NPC. EBV-miR-BART-22 displayed much stronger regulation of the nuclear translocation of β-catenin in NPC (Fig. 6C). In addition to EBV miRNA, other EBV genome or virus gene products also activate the Wnt pathway. For example, in nasopharyngeal cells infected with EBV, LMP1 induces the expression of β-catenin, thus potentially contributing to the malignant growth of NPC [48]. Therefore, various factors may affect the expression of these Wnt signaling-associated proteins, thereby affecting Wnt signaling correlation with EBV-miR-BART-22 in NPC. Furthermore, these results based on the prediction of bioinformatics analysis deserve our attention because some BART miRNAs (BART13 and BART19-3p) may regulate the Wnt/β-catenin pathway [49]. In a previous study, EBV-miR-BART22 was found to activate the PI3K/AKT and GSK3β/β-catenin pathways and their downstream tumor stemness and EMT signals [37]. Moreover, EBV-miR-BART22 induces miR-4721 expression through the PI3K/AKT/c-JUN/Sp1 signaling axis [50]. However, there are other molecules involved in NPC recurrence and metastasis of EBV-miR-BART22 that have yet to be identified. To further understand the role of EBV-miR-BART22 in NPC recurrence and metastasis. We found a new way that EBV-miR-BART-22 regulates the Wnt/β-catenin pathway (Fig. 6A, B). Our findings are different from those of previous studies showing that EBV-miR-BART22 may act as an enhancer factor rather than an initiator factor in NPC progression. We identified a new target and mechanism of EBV-miR-BART-22 that has not been reported in the past. The study of the pathway linking EBV-miR-BART-22 and Wnt/β-catenin is still worth further exploration to clarify the related underlying molecular mechanism.

To reveal the potential target genes of EBV-miR-BART-22 in NPC metastasis, we utilized RNA deep sequencing, a literature search, bioinformatics prediction, and luciferase reporter gene detection. We identified 3645 downregulated genes and many important pathways correlated with EBV-miR-BART-22 (Fig. 7A). Finally, we found that MOSPD2 is the primary cellular target of EBV-miR-BART-22 in NPC (Fig. 7B). More surprisingly, the expression of MOSPD2 was significantly reduced in NPCs compared with NPs (Fig. 7D). Motile sperm domain-containing protein 2 (MOSPD2) is a single-pass membrane protein to which no function was ascribed until recently [51, 52]. From the molecular mechanism, MOSPD2 has been shown to hijack resources from the host cell by promoting the formation of MCSs by VAP-A/VAP-B/MOSPD2 [53, 54]. By tethering the ER and other organelles, MOSPD2 most likely functions in intracellular exchanges and communication. MOSPD2, which is expressed on the plasma membrane of human monocytes, was explored for its potential to regulate cancer cell migration and metastasis [55, 56]. In our research, MOSPD2 had low expression in EBV-miR-BART-22 overexpressing cells. MOSPD2 is a beneficial protein in NPC, and this finding is in contrast to that of a previous study of cancer showing that MOSPD2 promotes cancer migration. EBV-miR-BART-22 targets MOSPD2 in NPC. The posttranscriptional regulation of miRNA is complex, as a single miRNA could target multiple mRNAs, while multiple miRNAs could target a single mRNA [57, 58]. Due to the multiple targets a miRNA could have, it is difficult to predict all possible targets of each miRNA [59, 60]. Although in the present research we identified MOSPD2 as an EBV-miR-BART-22 target in NPC cells, further study is still required to verify the correlation between MOSPD2 and EBV-miR-BART-22 expression and eventually correlate MOSPD2 expression with the clinical data in the tissue microarray containing the same specimens. We also did not detect the combination of MOSPD2 with β­catenin based on exogenous Co-IP. Therefore, it is likely that many targets of EBV-miR-BART-22 in NPC remain to be elucidated.

In conclusion, our results showed that EBV-miR-BART-22 plays an oncogenic role by driving the EMT and metastasis of NPC by targeting MOSPD2 and activating the Wnt/β-catenin pathway. Thus, EBV-miR-BART-22 is a potential prognostic biomarker and novel antimetastatic target for NPC.

## Supplementary information

Original Data File

reproducibility checklist

Supplementary Figure

Supplementary Table

Animal Studies Ethics Committee Approval

Ethics Committee Approval and Patient Consent

## Data Availability

Public data used in this work can be acquired from the Gene Expression Omnibus (GEO, http://www.ncbi.nlm.nih.gov/geo/). The raw experimental data and analysis codes supporting the conclusions of this article will be made available by the corresponding author.
