# Supplementary material for: Epstein–Barr virus-encoded microRNA BART22 serves as novel biomarkers and drives malignant transformation of nasopharyngeal carcinoma
Source: Cell Death Dis. 2022 Jul 30;13(7):664. doi: 10.1038/s41419-022-05107-x (PMC9338958; doi:10.1038/s41419-022-05107-x)

Figure 4 H

CNE-1 GAPDH

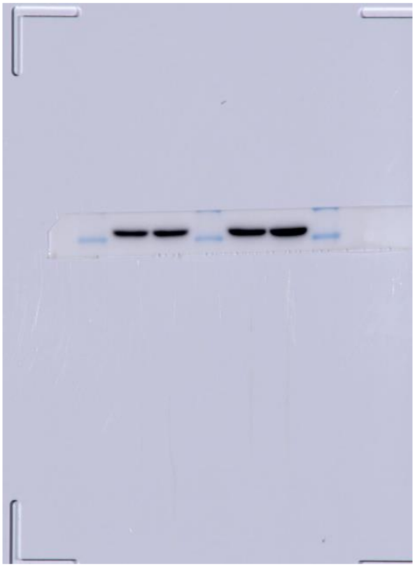

CNE-1 Snail

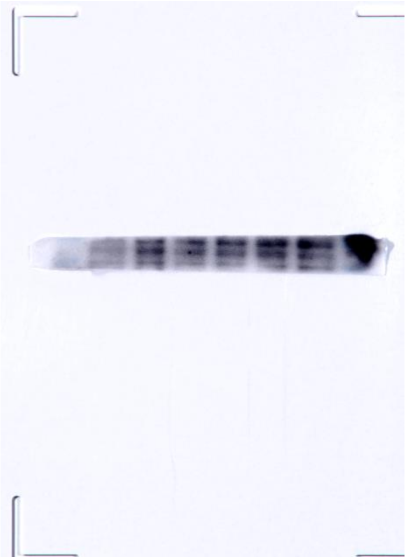

CNE-1 Vimentin

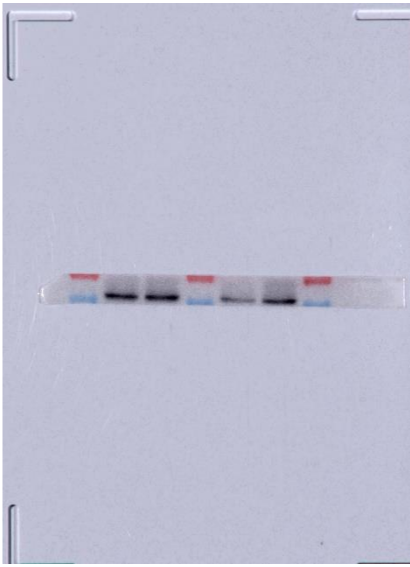

CNE-1 E-cadherin

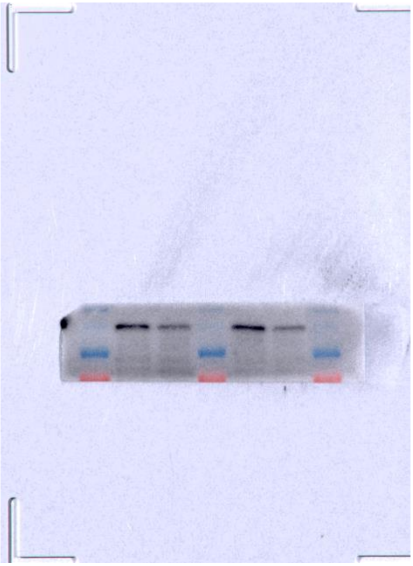

CNE-1 N-cadherin

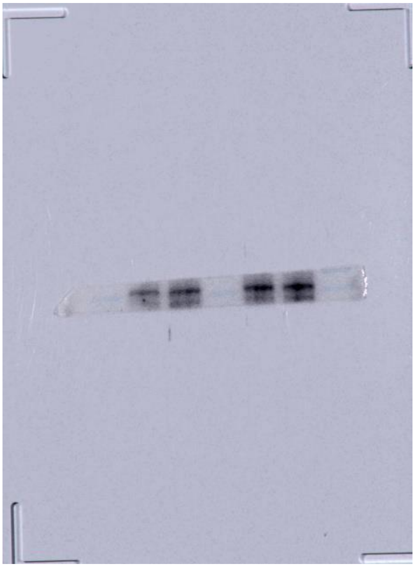

Figure 4 H

CNE-2 GAPDH

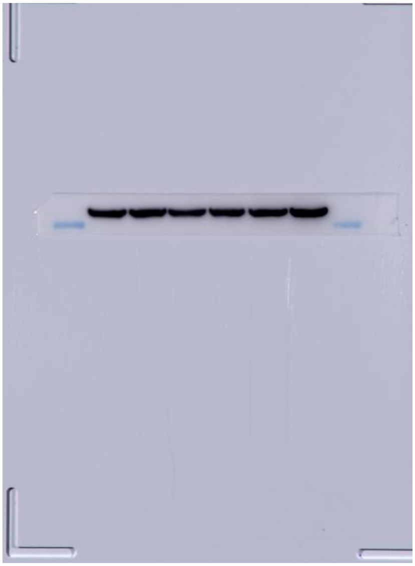

CNE-2 Snail

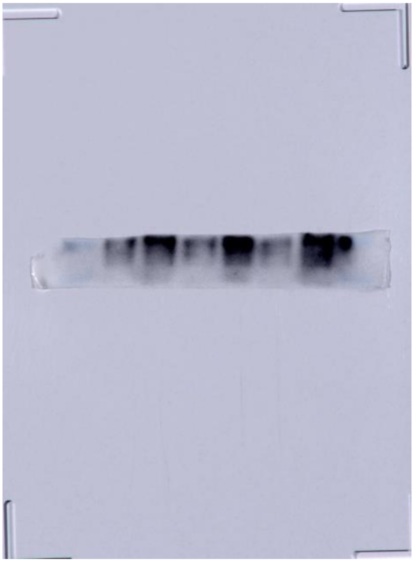

CNE-2 Vimentin

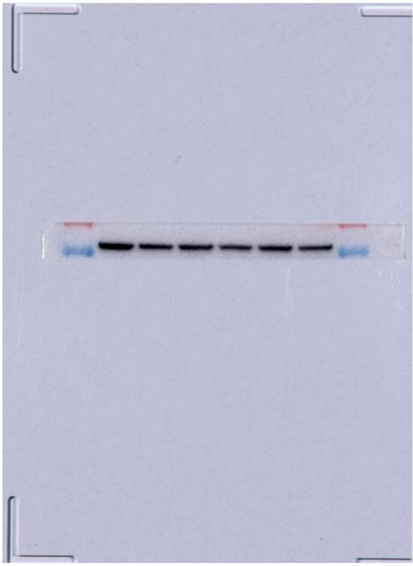

CNE-2 E-cadherin

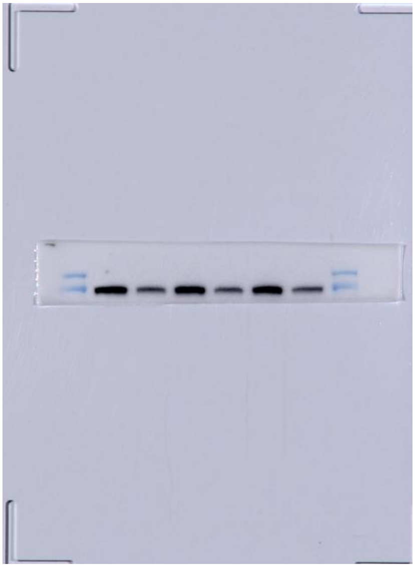

CNE-2 N-cadherin

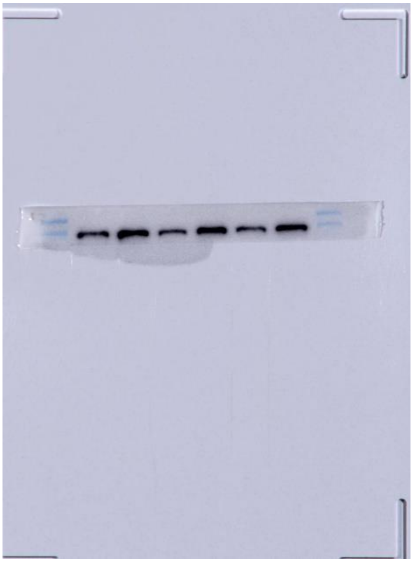

Figure 4 H

SUNE-1 GAPDH

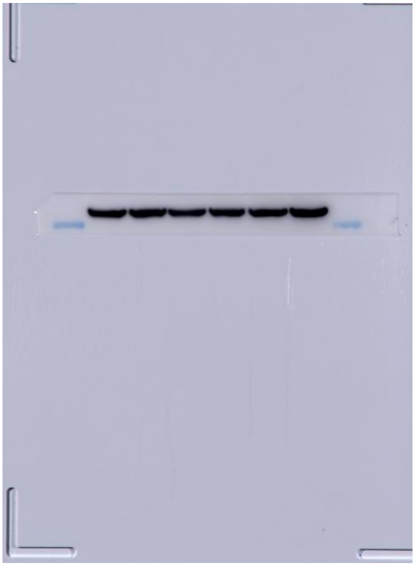

SUNE-1 Snail

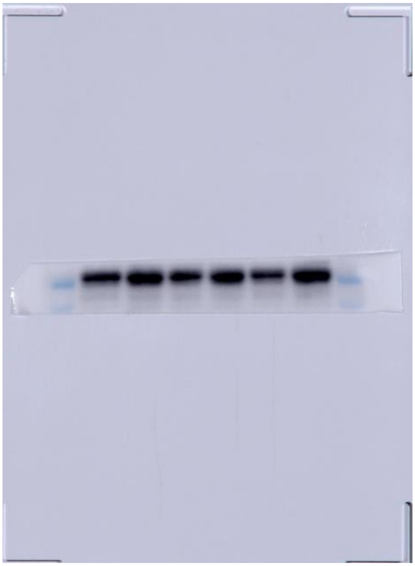

SUNE-1 Vimentin

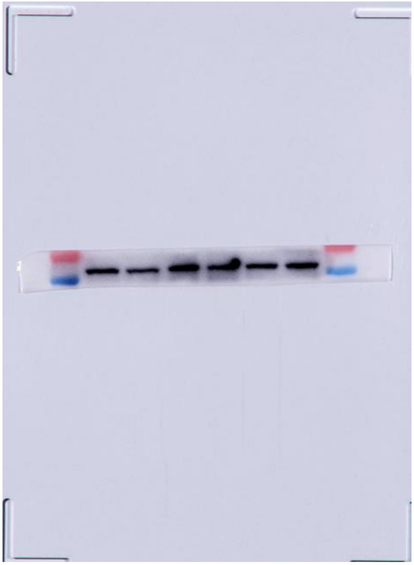

SUNE-1 E-cadherin

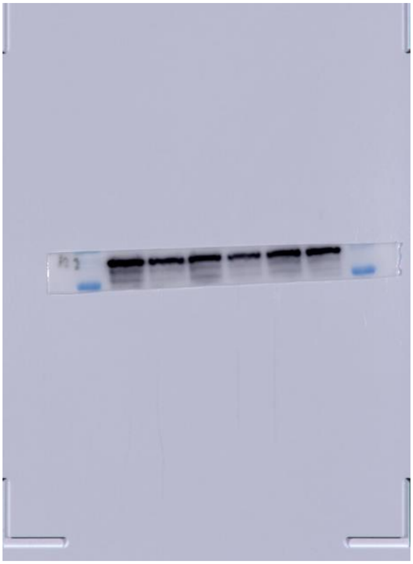

SUNE-1 N-cadherin

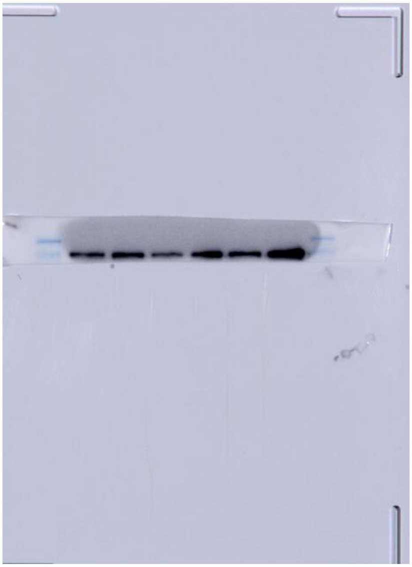

Figure 5 G

C666-1 GAPDH

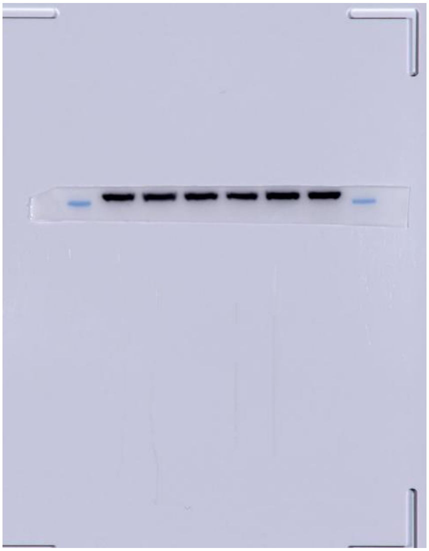

C666-1 Snail

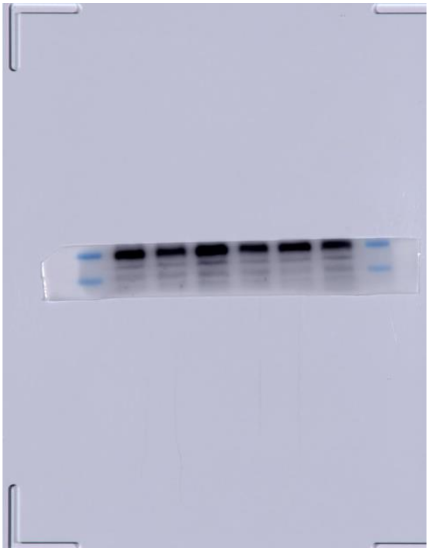

C666-1 Vimentin

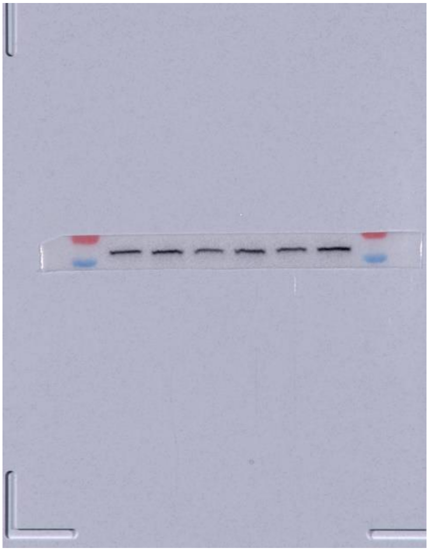

C666-1 E-cadherin

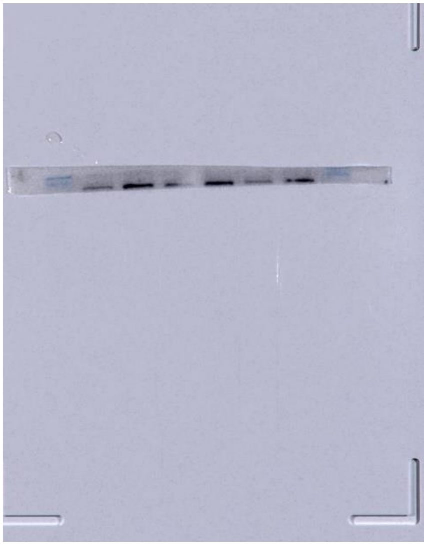

C666-1 N-cadherin

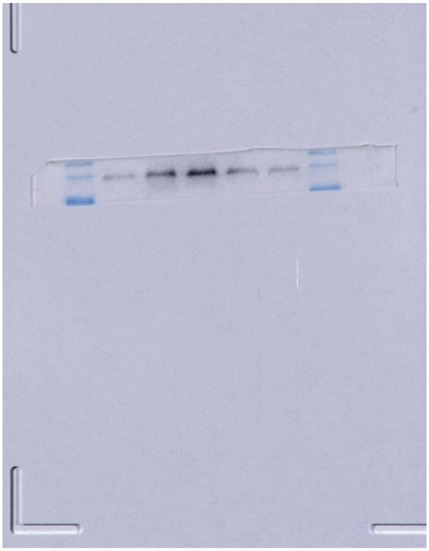

Figure 6 C

CNE-1   Histone3

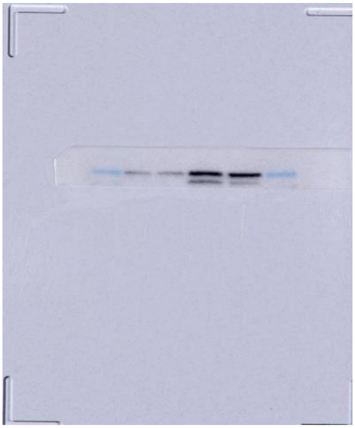

CNE-1   GAPDH

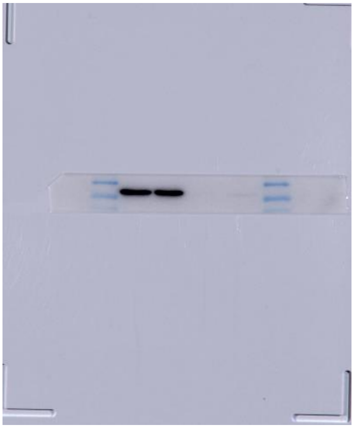

CNE-1   β-catenin

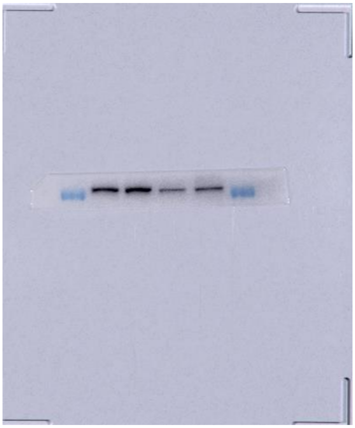

CNE-2   Histone3

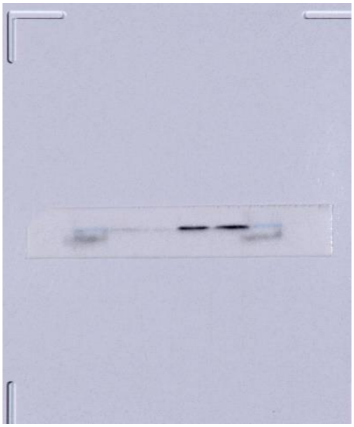

CNE-2   GAPDH

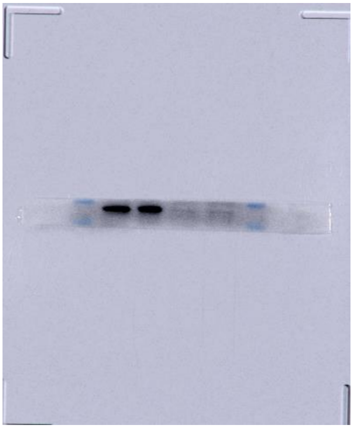

CNE-2   β-catenin

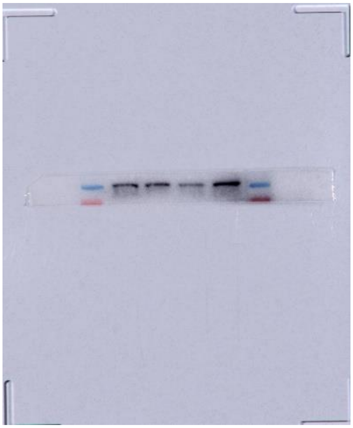

CNE-2   Histone3

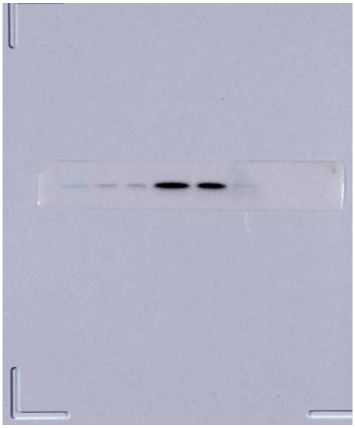

CNE-2   GAPDH

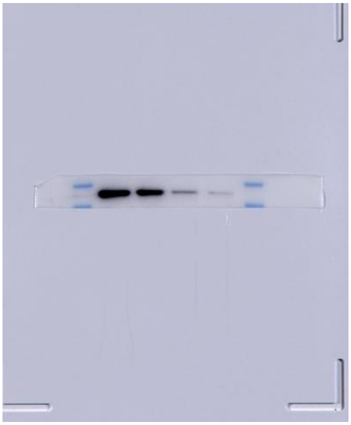

CNE-2   β-catenin

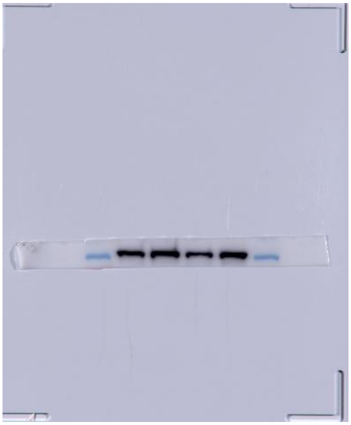

C666-1 Histone3

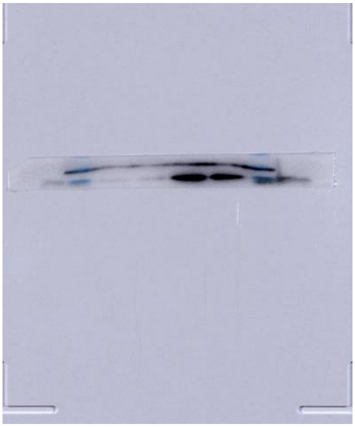

C666-1 GAPDH

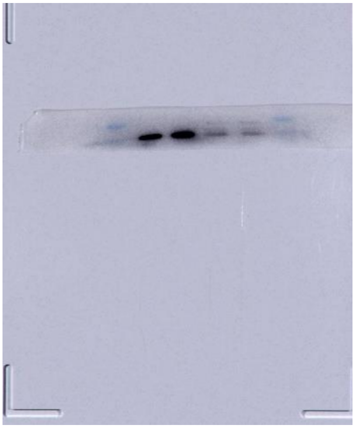

C666-1 β-catenin

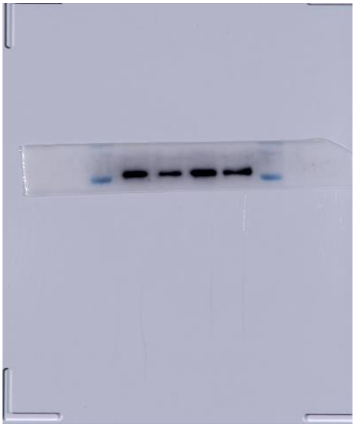

Figure 6 G

CNE-1 BART22 GAPDH

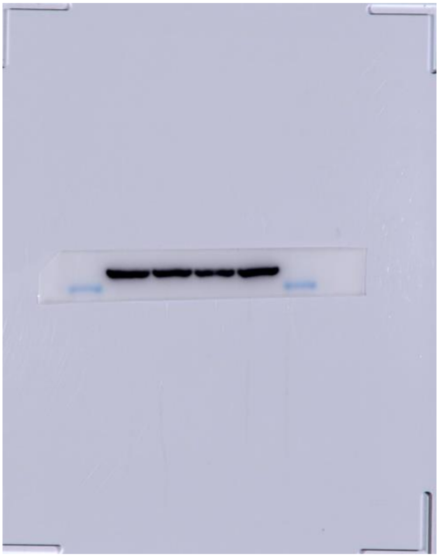

CNE-1 BART22 Snail

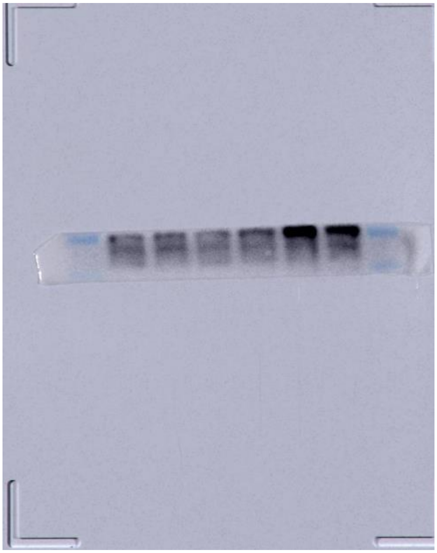

CNE-1 BART22 Vimentin

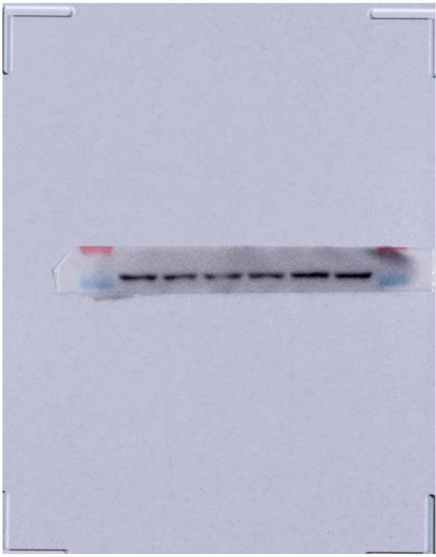

CNE-1 BART22 β-catenin

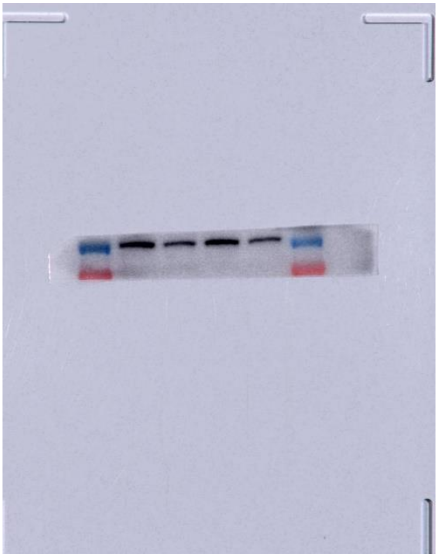

CNE-1 BART22 E-cadherin

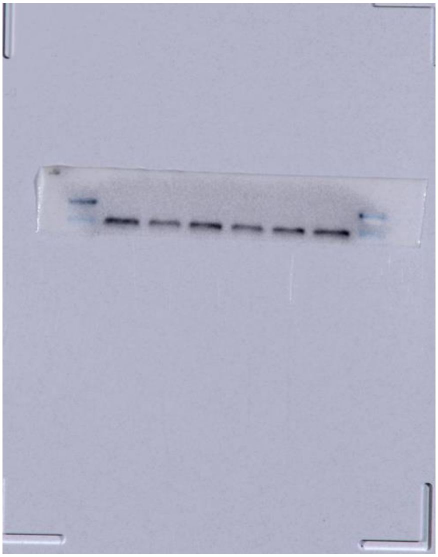

CNE-1 BART22 N-cadherin

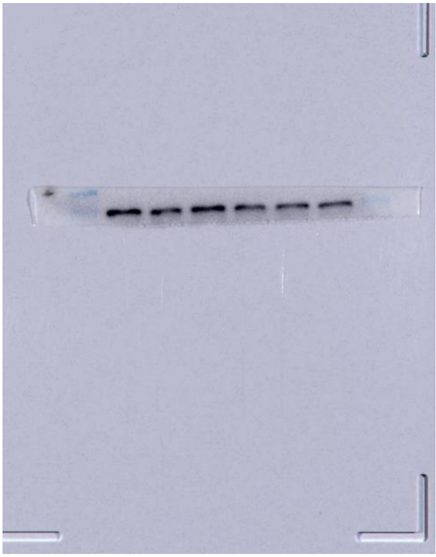

Figure 6 G

CNE-2 BART22 GAPDH

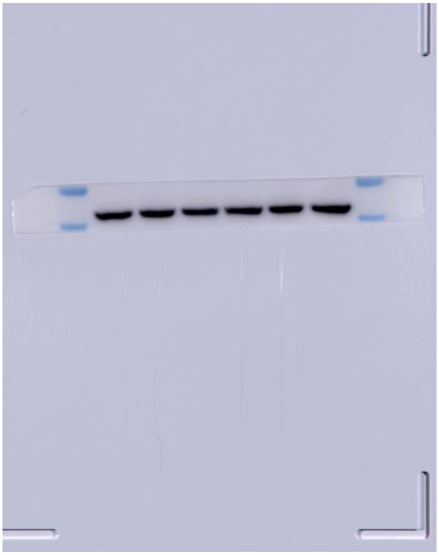

CNE-2 BART22 Snail

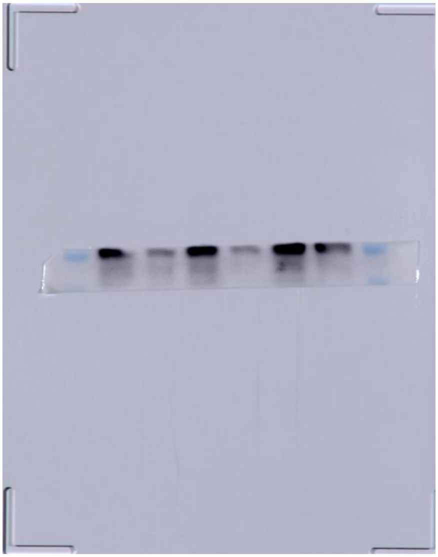

CNE-2 BART22 Vimentin

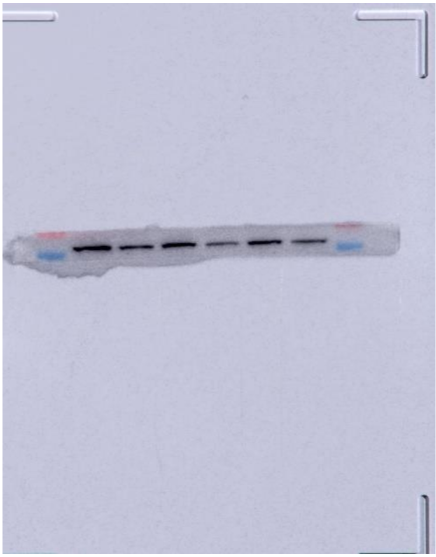

CNE-2 BART22 β-catenin

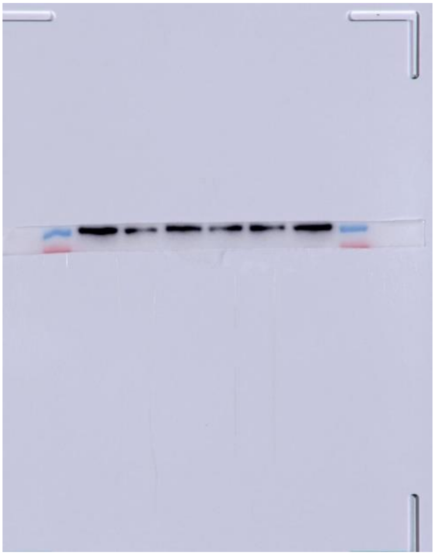

CNE-2 BART22 E-cadherin

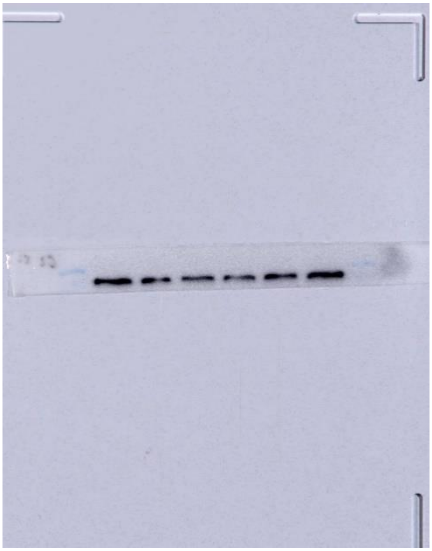

CNE-2 BART22 N-cadherin

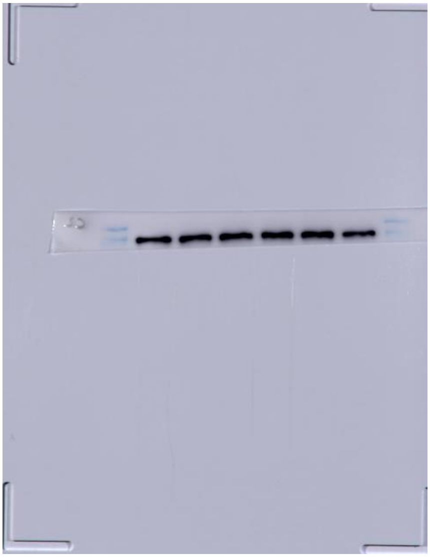

Figure 6 G

SUNE-1 BART22 GAPDH

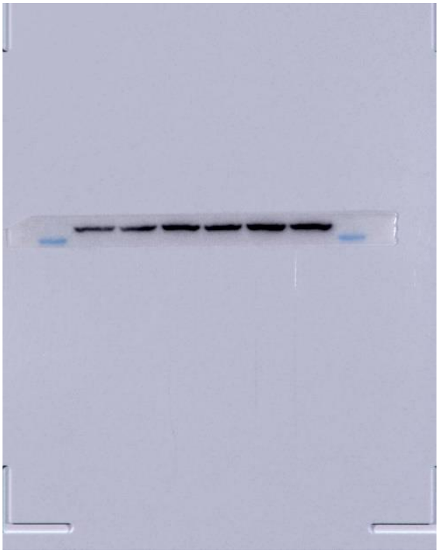

SUNE-1 BART22 Snail

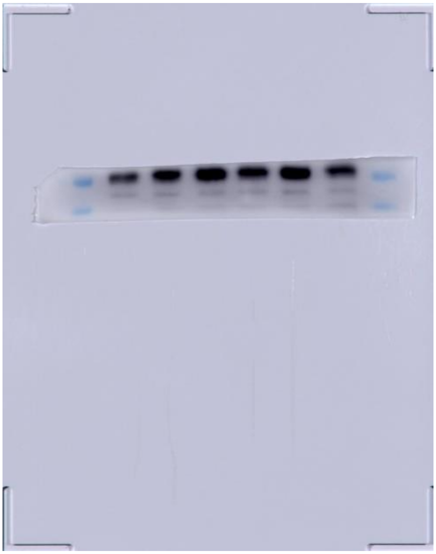

SUNE-1 BART22 Vimentin

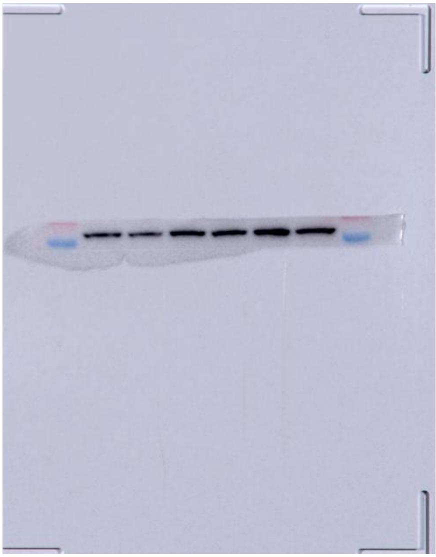

SUNE-1 BART22 β-catenin

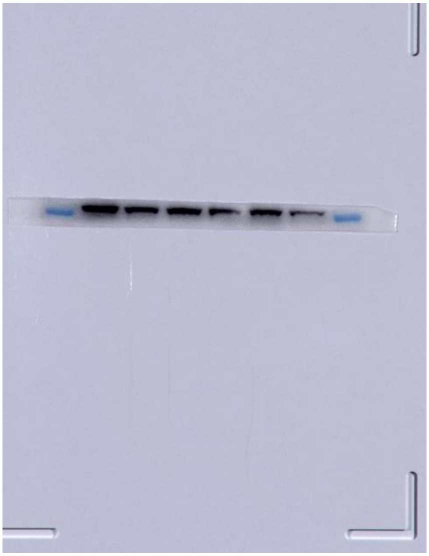

SUNE-1 BART22 E-cadherin

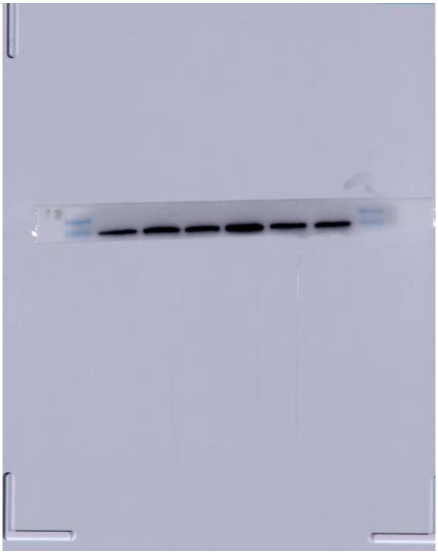

SUNE-1 BART22 N-cadherin

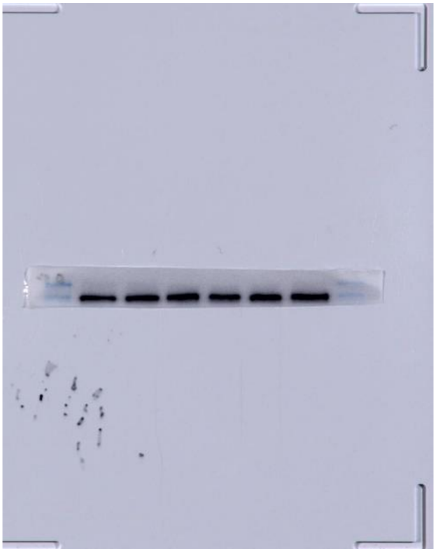

Figure 6 G

C666-1 shBART22 GAPDH

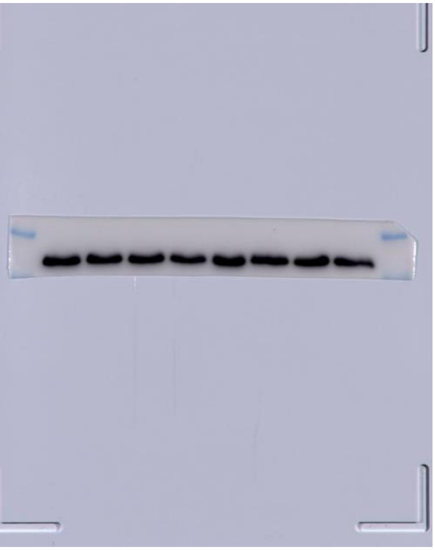

C666-1 shBART22 Snail

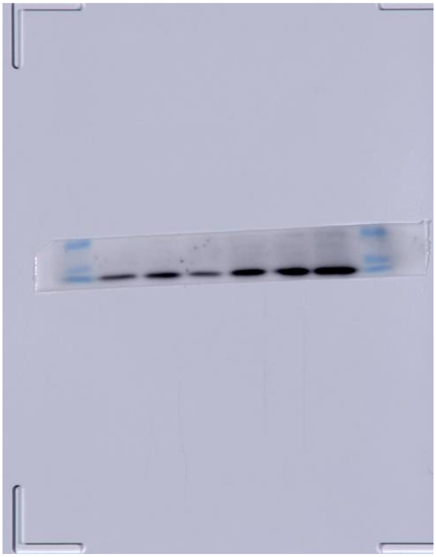

C666-1 shBART22 Vimentin

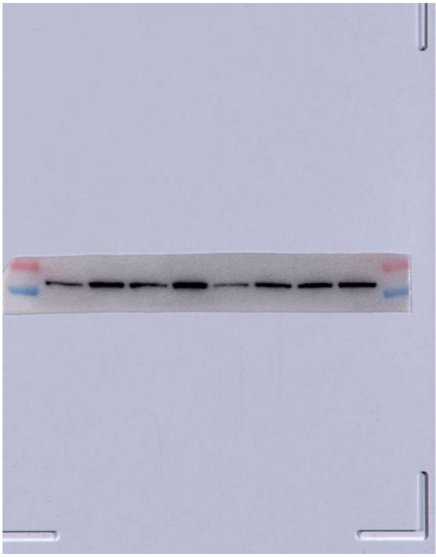

C666-1 shBART22 β-catenin

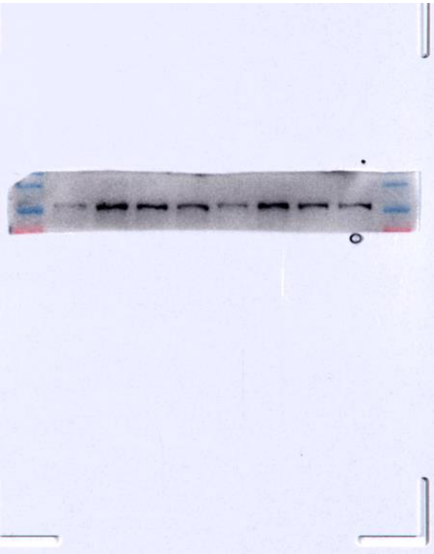

C666-1 shBART22 E-cadherin

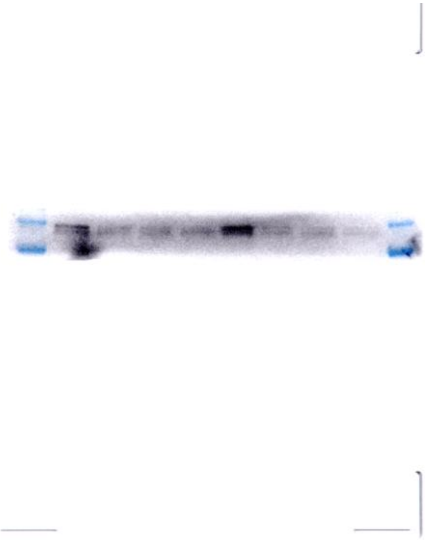

C666-1 shBART22 N-cadherin

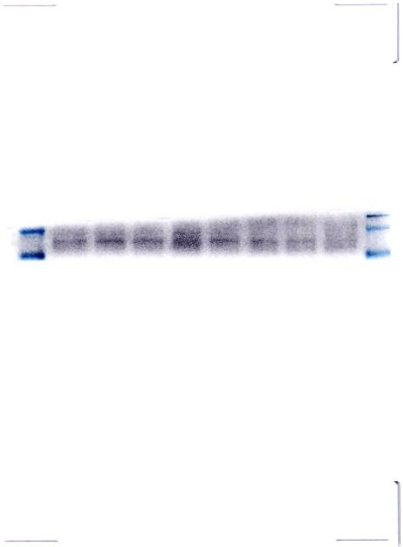

Figure 7 C

CNE-1 GAPDH

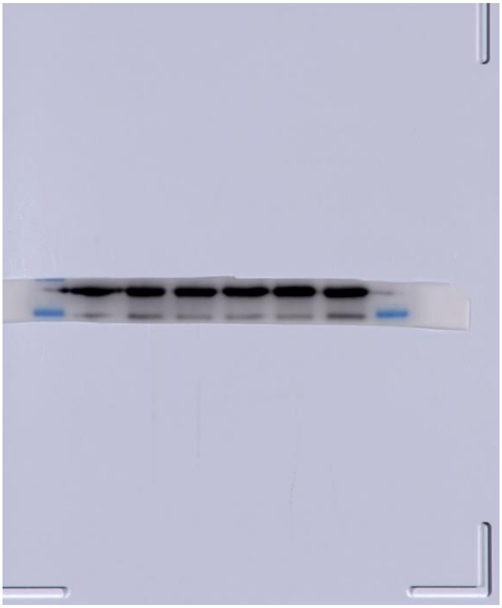

CNE-1 MOSPD2

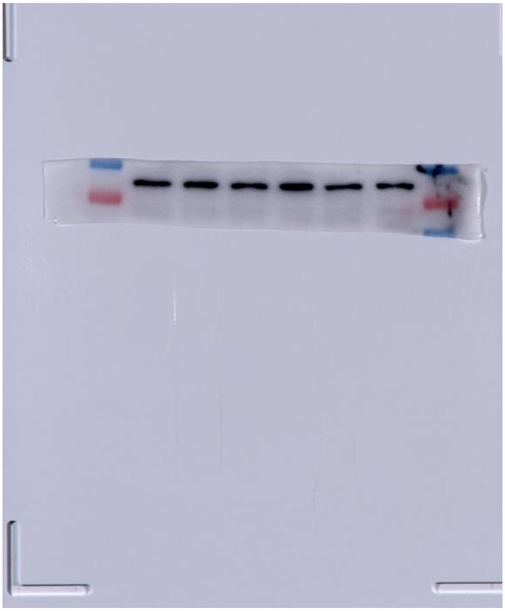

CNE-2 GAPDH

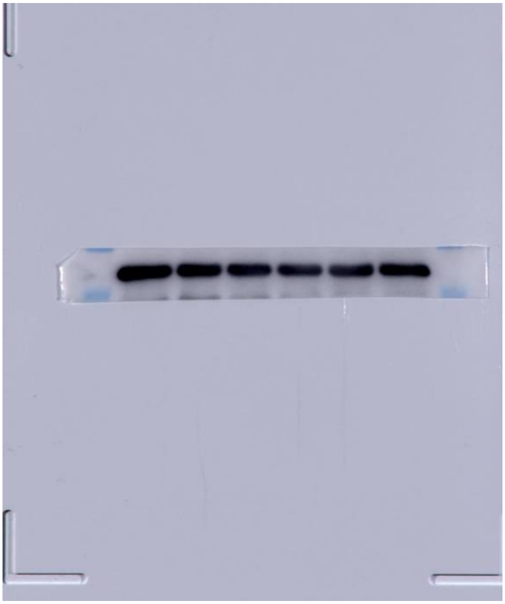

CNE-2 MOSPD2

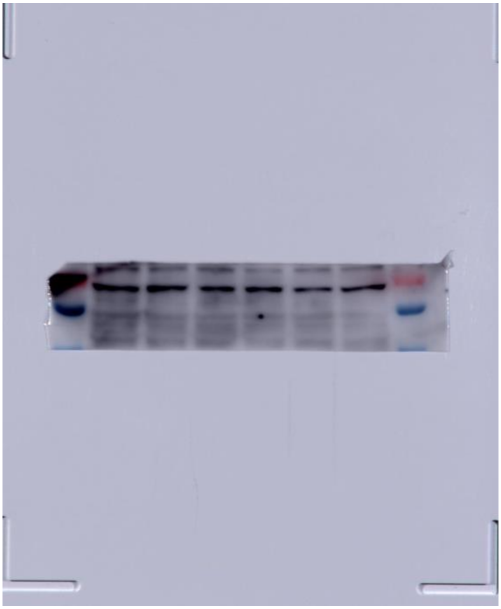

Figure 7 C

SUNE-1 GAPDH

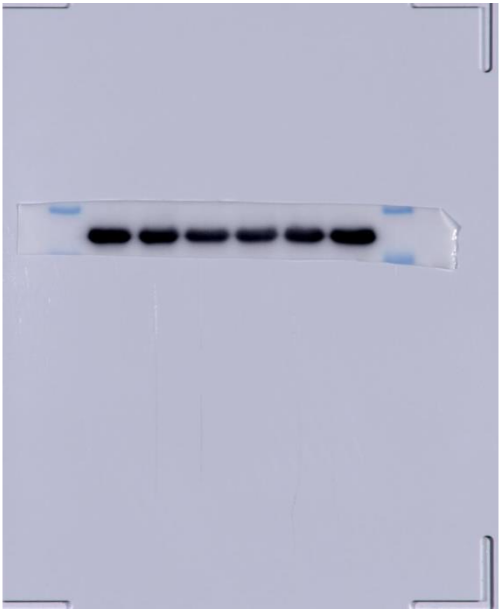

SUNE-1 MOSPD2

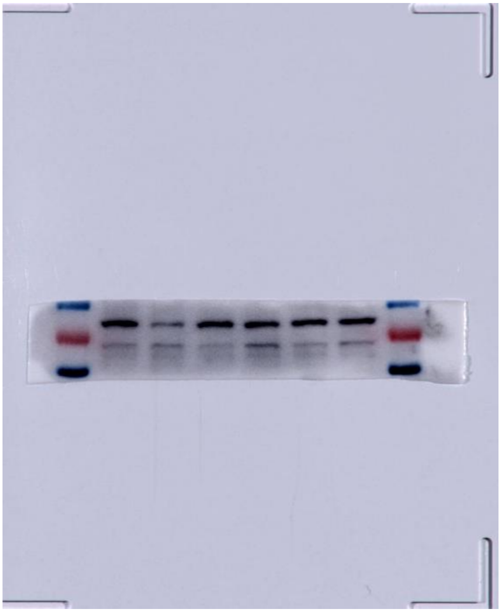

C666-1 GAPDH

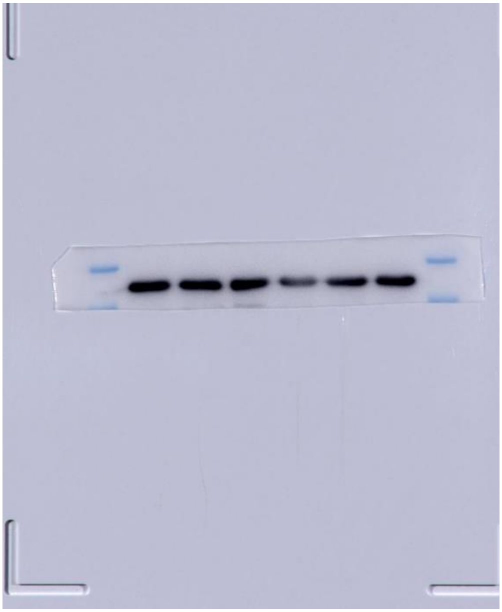

C666-1 MOSPD2

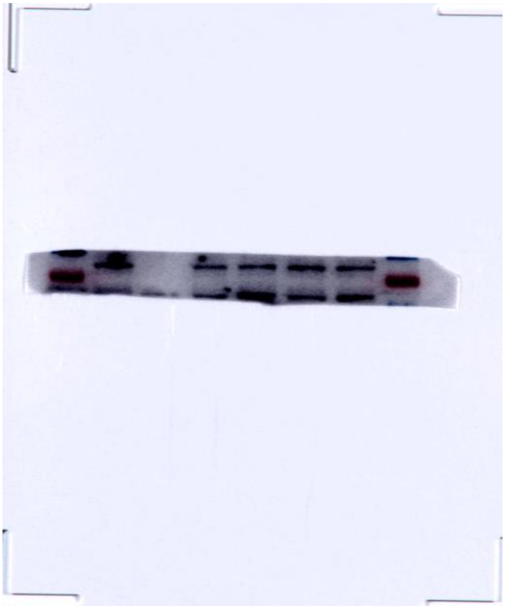

Figure 7 J

CNE-1 BART22 GAPDH

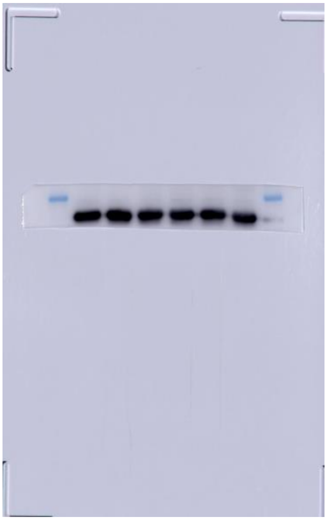

CNE-1 BART22 MOSPD2

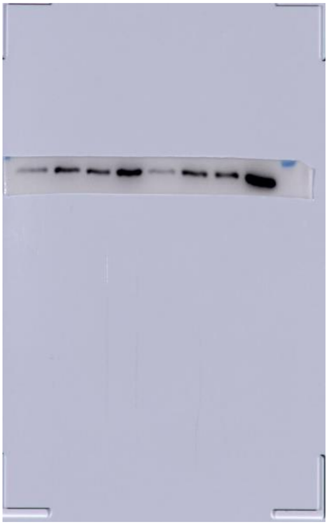

CNE-1 BART22 Snail

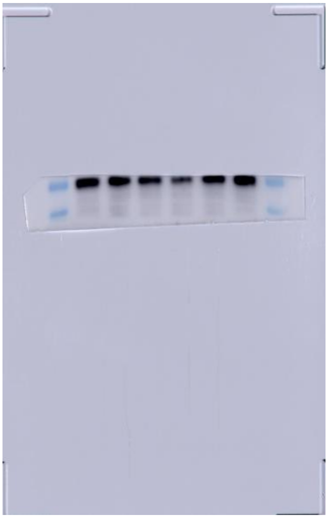

CNE-1 BART22 Vimentin

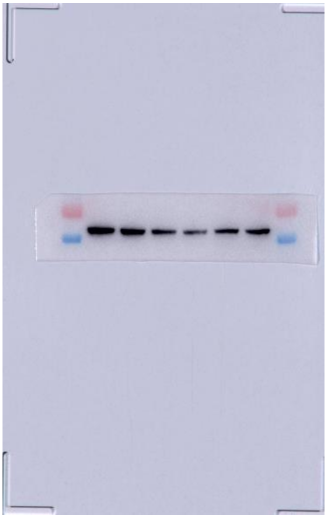

CNE-1 BART22 β-catenin

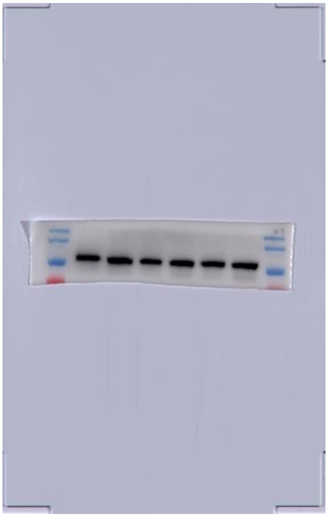

CNE-1 BART22 E-cadherin

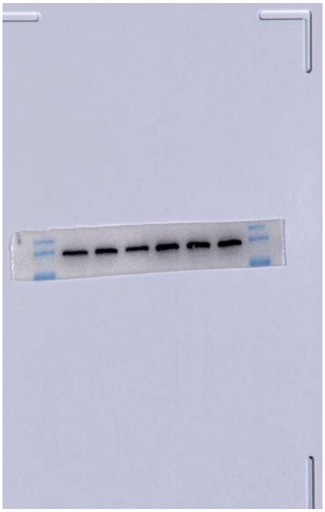

CNE-1 BART22 N-cadherin

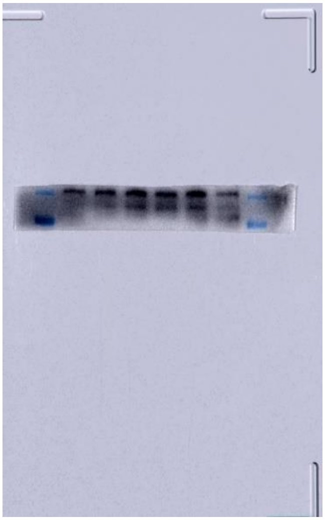

Figure 7 J

CNE-2 BART22 GAPDH

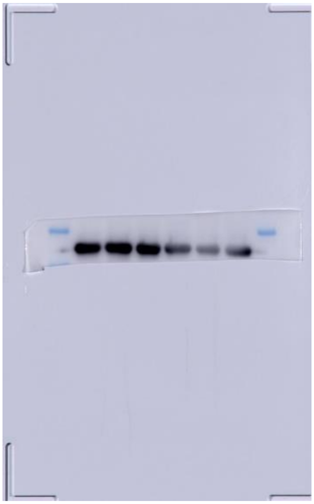

CNE-2 BART22 MOSPD2

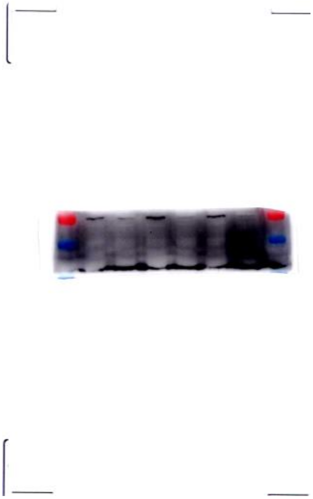

CNE-2 BART22 Snail

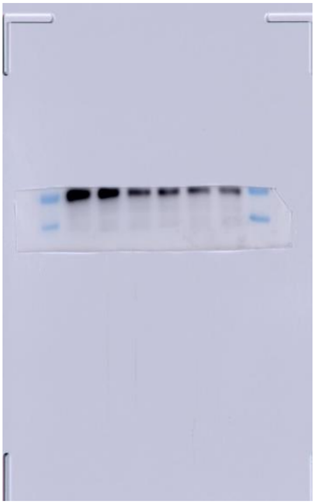

CNE-2 BART22 Vimentin

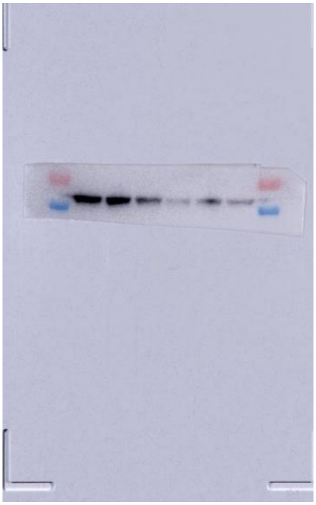

CNE-2 BART22 β-catenin

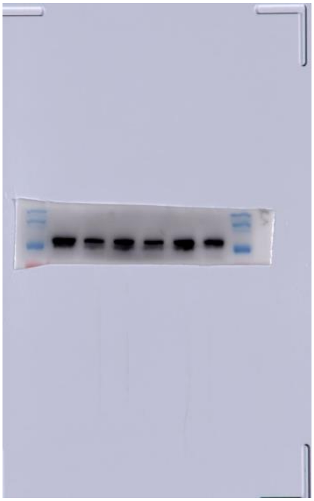

CNE-2 BART22 E-cadherin

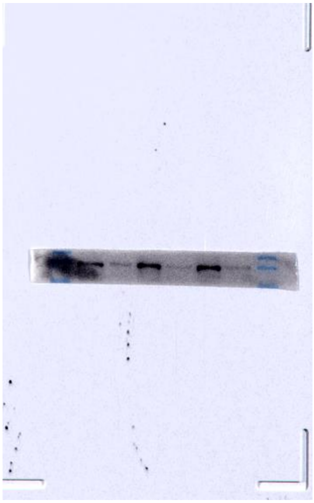

CNE-2 BART22 N-cadherin

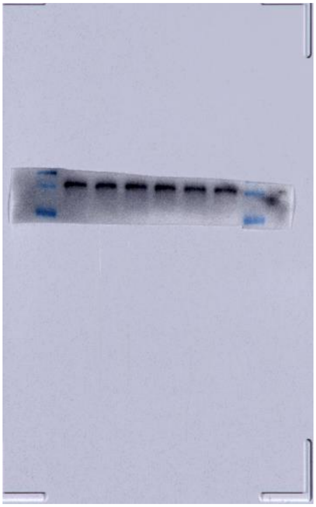

Figure 7 J

SUNE-1 BART22 GAPDH

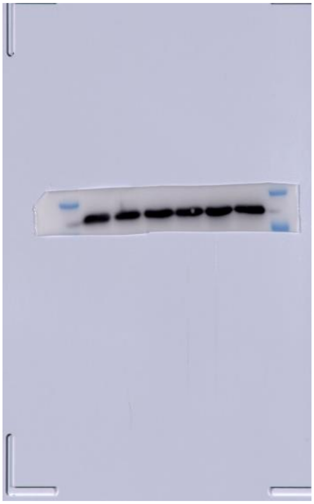

SUNE-1 BART22 MOSPD2

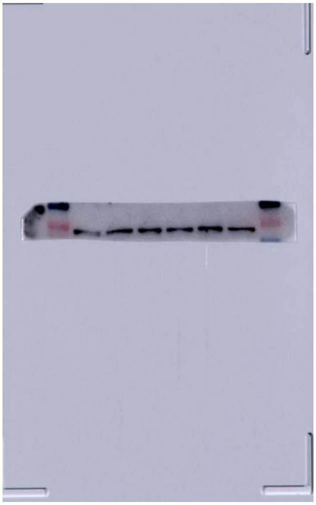

SUNE-1 BART22 Snail

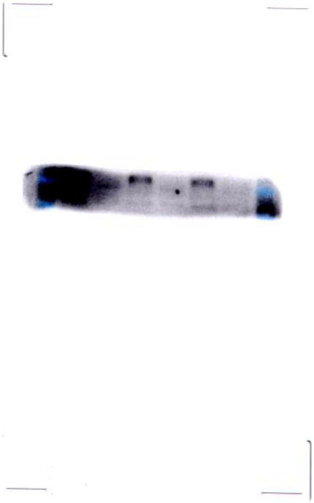

SUNE-1 BART22 Vimentin

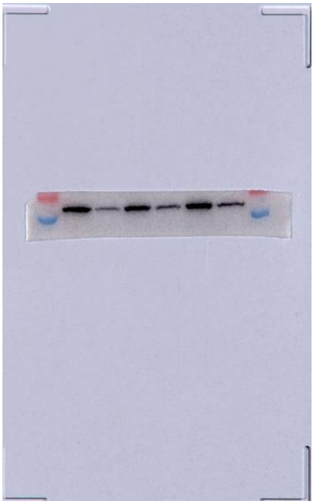

SUNE-1 BART22 β-catenin

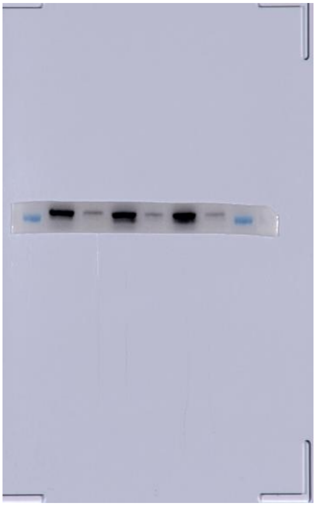

SUNE-1 BART22 E-cadherin

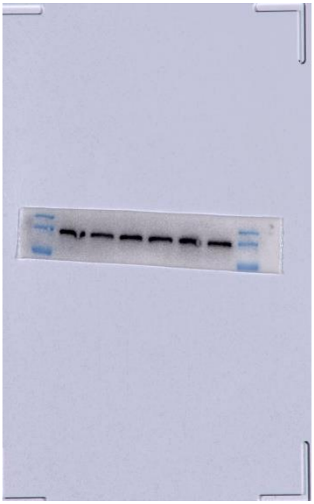

SUNE-1 BART22 N-cadherin

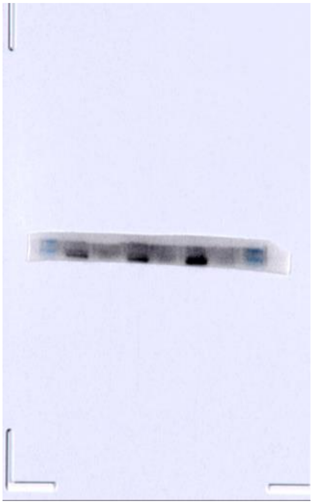

Figure 7 J

C666-1 shBART22 GAPDH

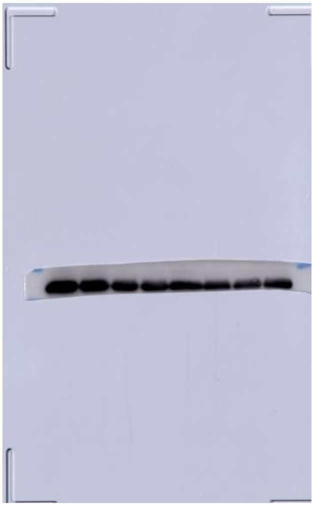

C666-1 shBART22 MOSPD2

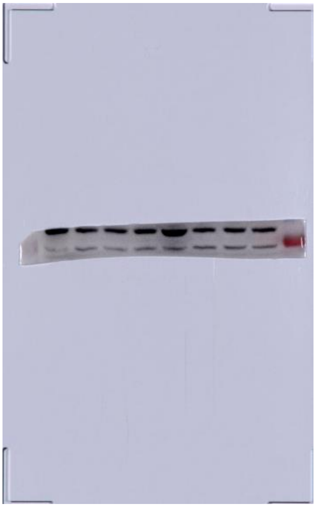

C666-1 shBART22 Snail

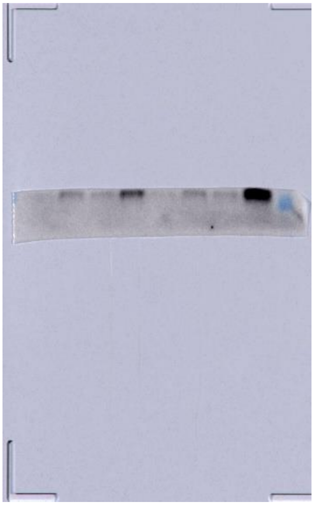

C666-1 shBART22 Vimentin

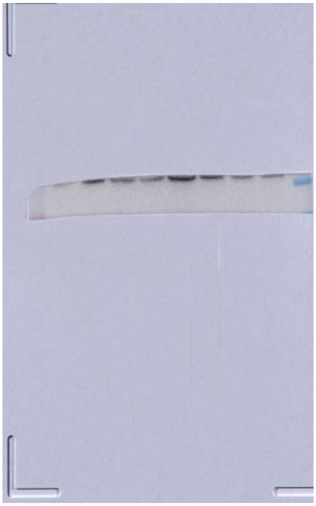

C666-1 shBART22 β-catenin

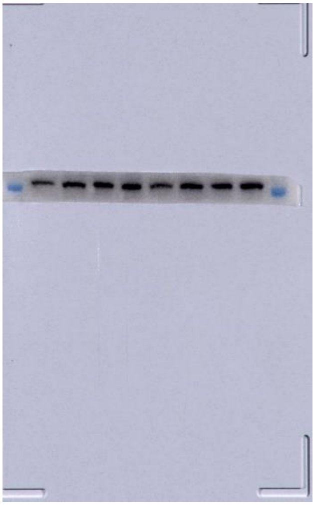

C666-1 shBART22 E-cadherin

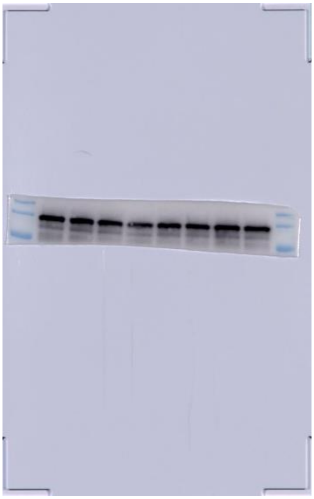

C666-1 shBART22 N-cadherin

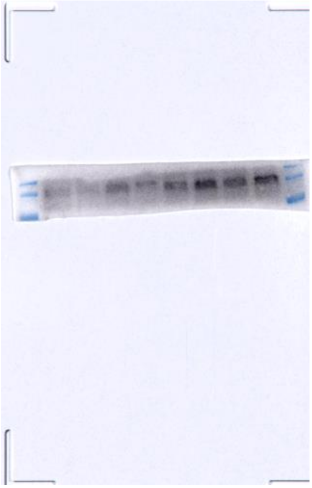

Supplement: Supplementary file 1 — Original Data File [file 41419_2022_5107_MOESM1_ESM.pdf]
