# Supplementary material for: Epstein–Barr virus-encoded microRNA BART22 serves as novel biomarkers and drives malignant transformation of nasopharyngeal carcinoma
Source: Cell Death Dis. 2022 Jul 30;13(7):664. doi: 10.1038/s41419-022-05107-x (PMC9338958; doi:10.1038/s41419-022-05107-x)
Supplement: Supplementary file 3 — Supplementary Figure [file 41419_2022_5107_MOESM3_ESM.pdf]

Supplementary 1

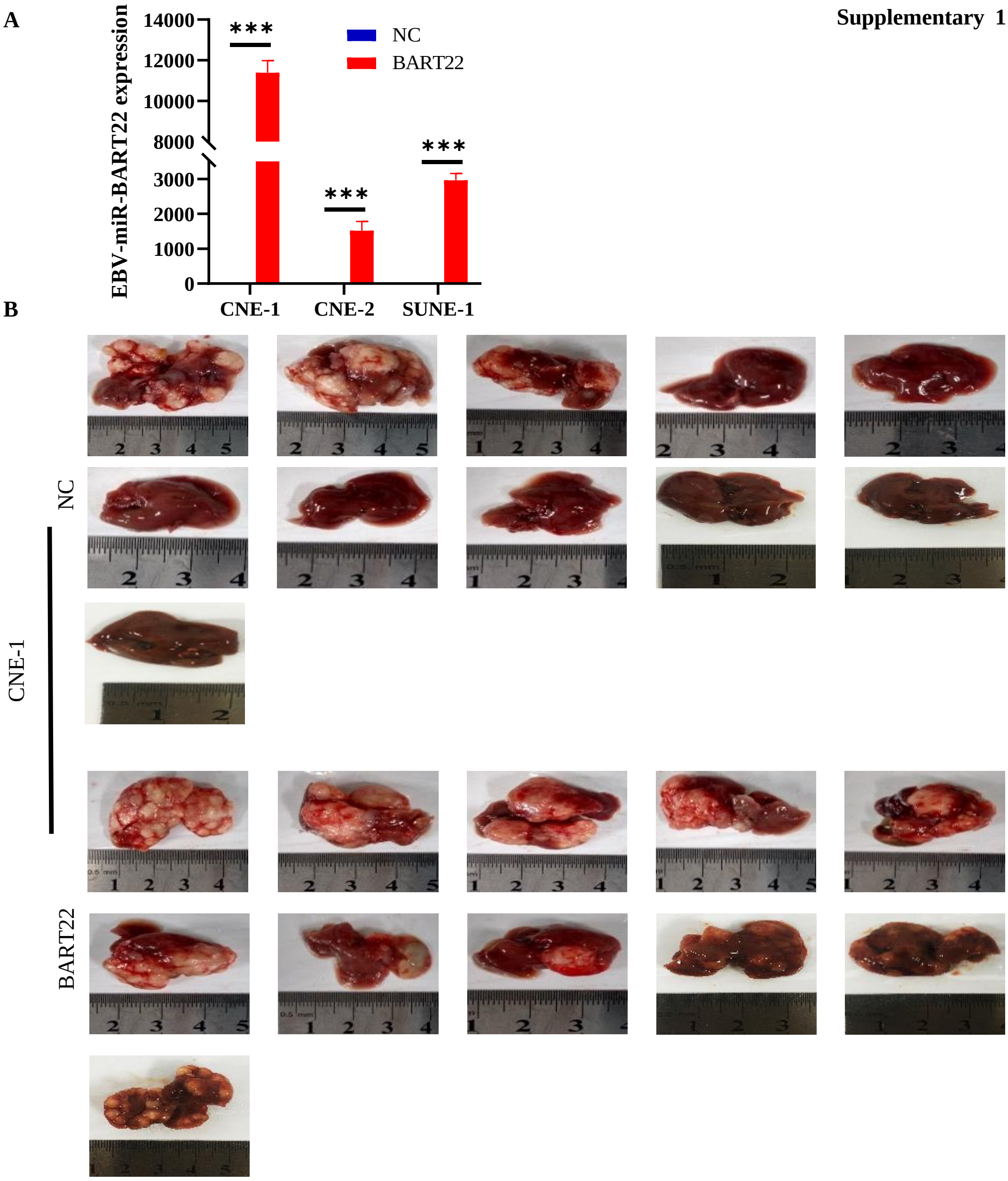

Supplementary Figure 1. Up-regulation of EBV-miR-BART-22 in EBV-negative NPC cells by lentivirus-mediated transduction. A. qPCR assay confirmed the up-regulation of EBV-miR-BART-22 in CNE-1 BATRT22, CNE-2 BART22 and SNUE-1 BART22 cells compared with relative mock control cells. B. Representative bright-field image of the livers was shown.

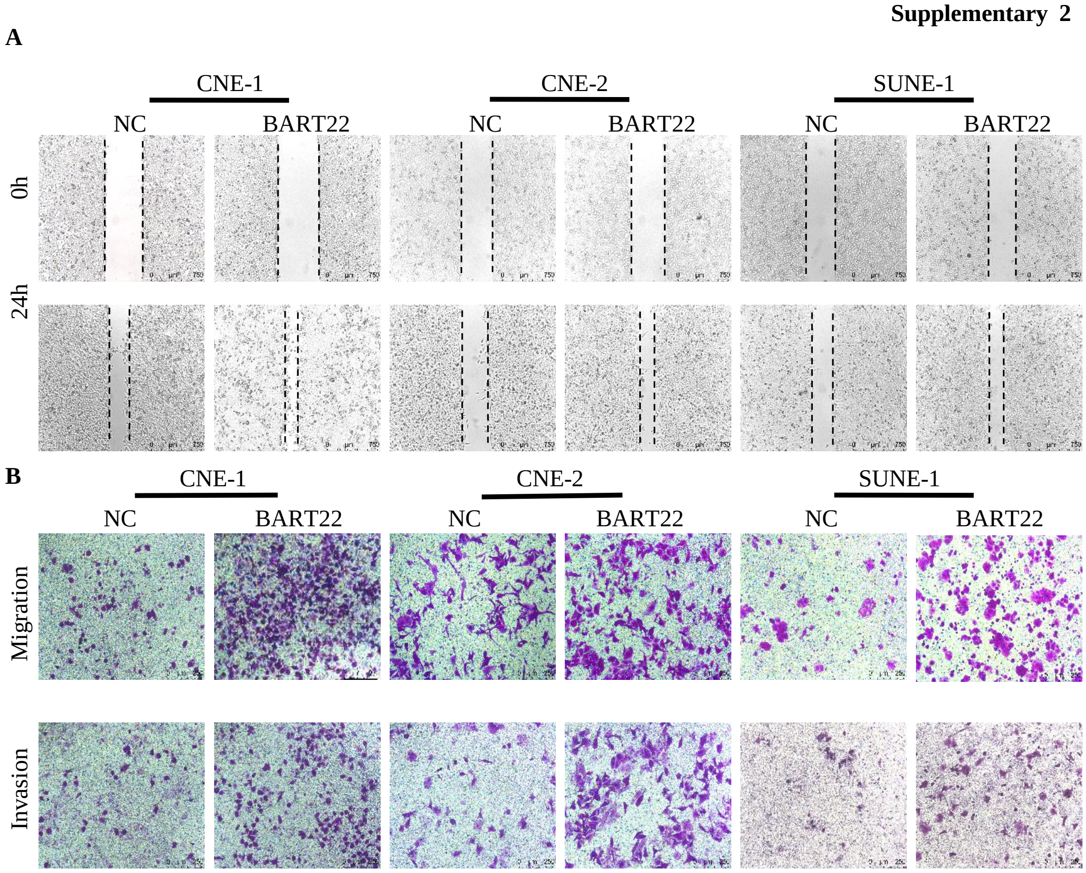

Supplementary Figure 7. Over-expression of EBV-miR-BART22 promotes the migration, invasion and EMT of NPC cells. A. Representative photomicrographs of scratch wounds at 0 and 24 h after wounds were made. B. Representative images and quantification of migration and invasion assays in CNE-1, CNE-2, SUNE-1 cells.

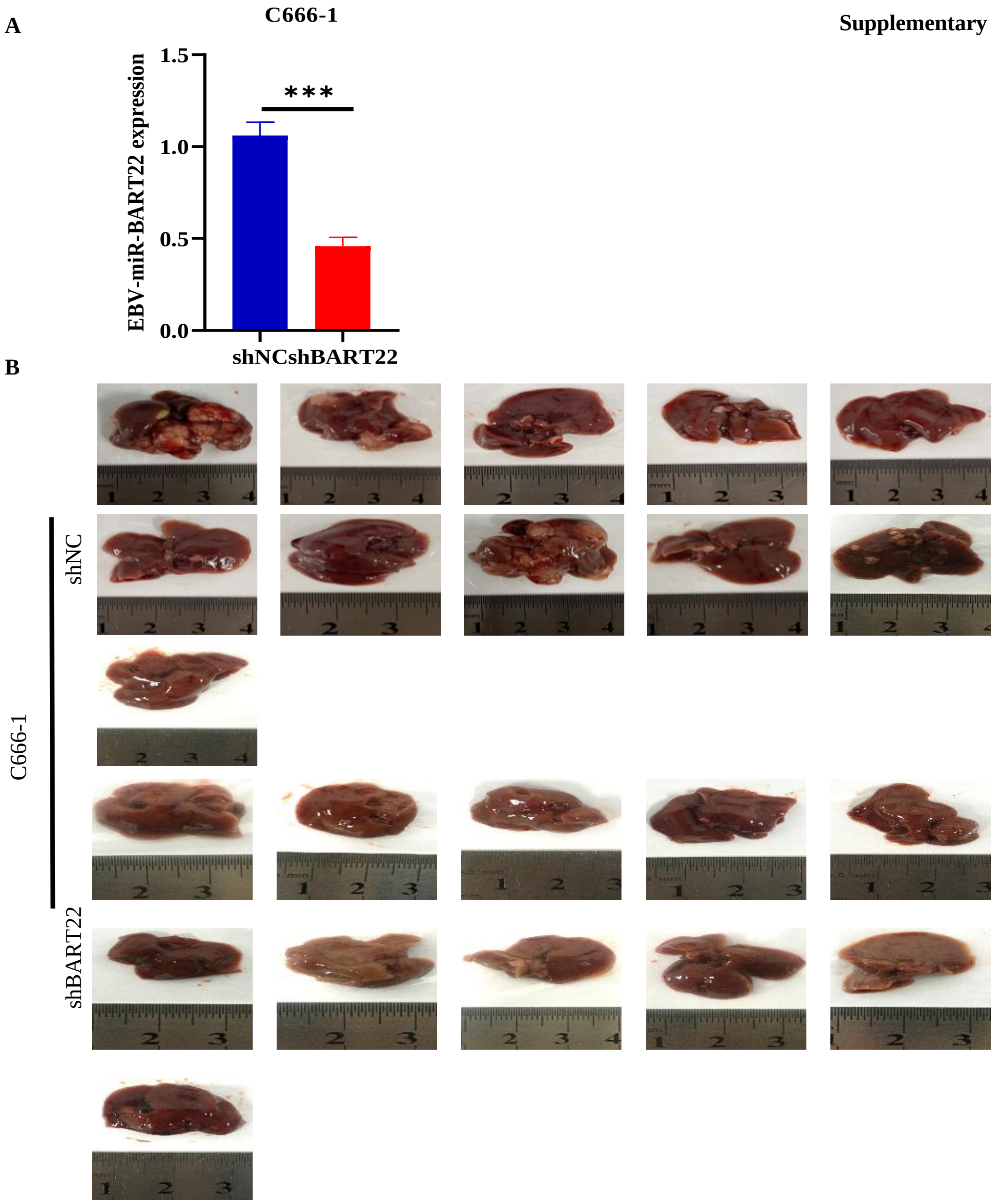

Supplementary Figure 3. Down-regulation of EBV-miR-BART-22 in EBV-positive NPC cells by lentivirus-mediated transduction. A. qPCR assay reveals the down-regulation of EBV-miR-BART-22 in C666-1 shBATRT22 in those transfected with control vectors. B. Representative bright-field image of the livers was shown

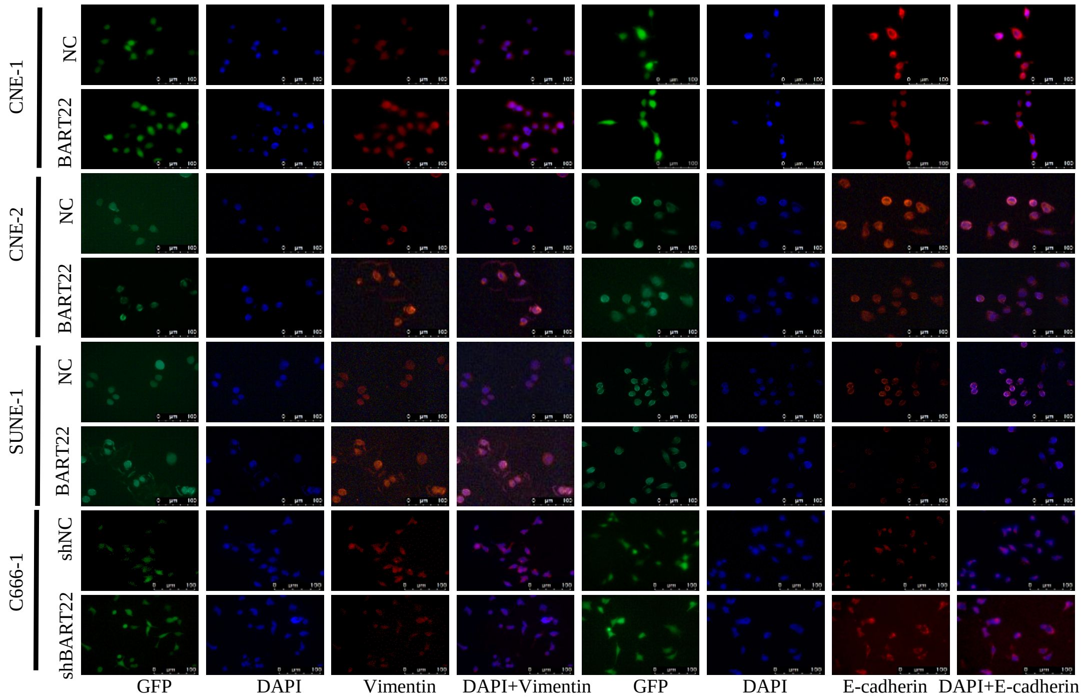

Supplementary Figure 4. EBV-miR-BART22 induces EMT of NPC cells in vitro. Immunofluorescence staining of E-cadherin and vimentin in indicated cells.

Supplementary 5

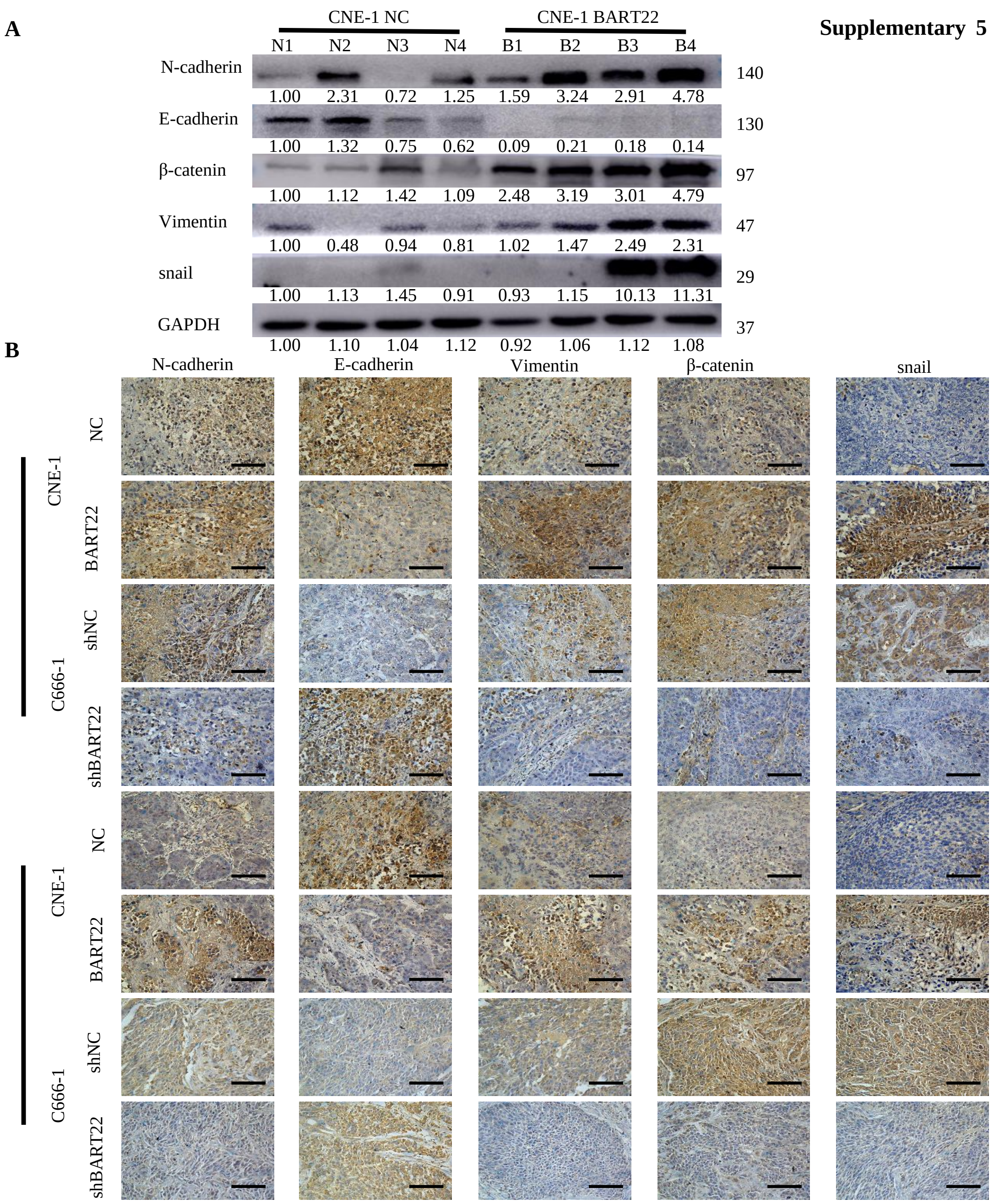

Supplementary Figure 5. EBV-miR-BART22 induces the change of EMT-related molecules in xenograft tissues. A. The expression of E-cadherin, N-cadherin, Vimentin, sanil and β-catenin was detected by western blotting in xenograft tissues. B. IHC analysis of E-cadherin, N-cadherin, Vimentin, sanil and β-catenin expression in liver metastasis tissues. Representative images are shown.

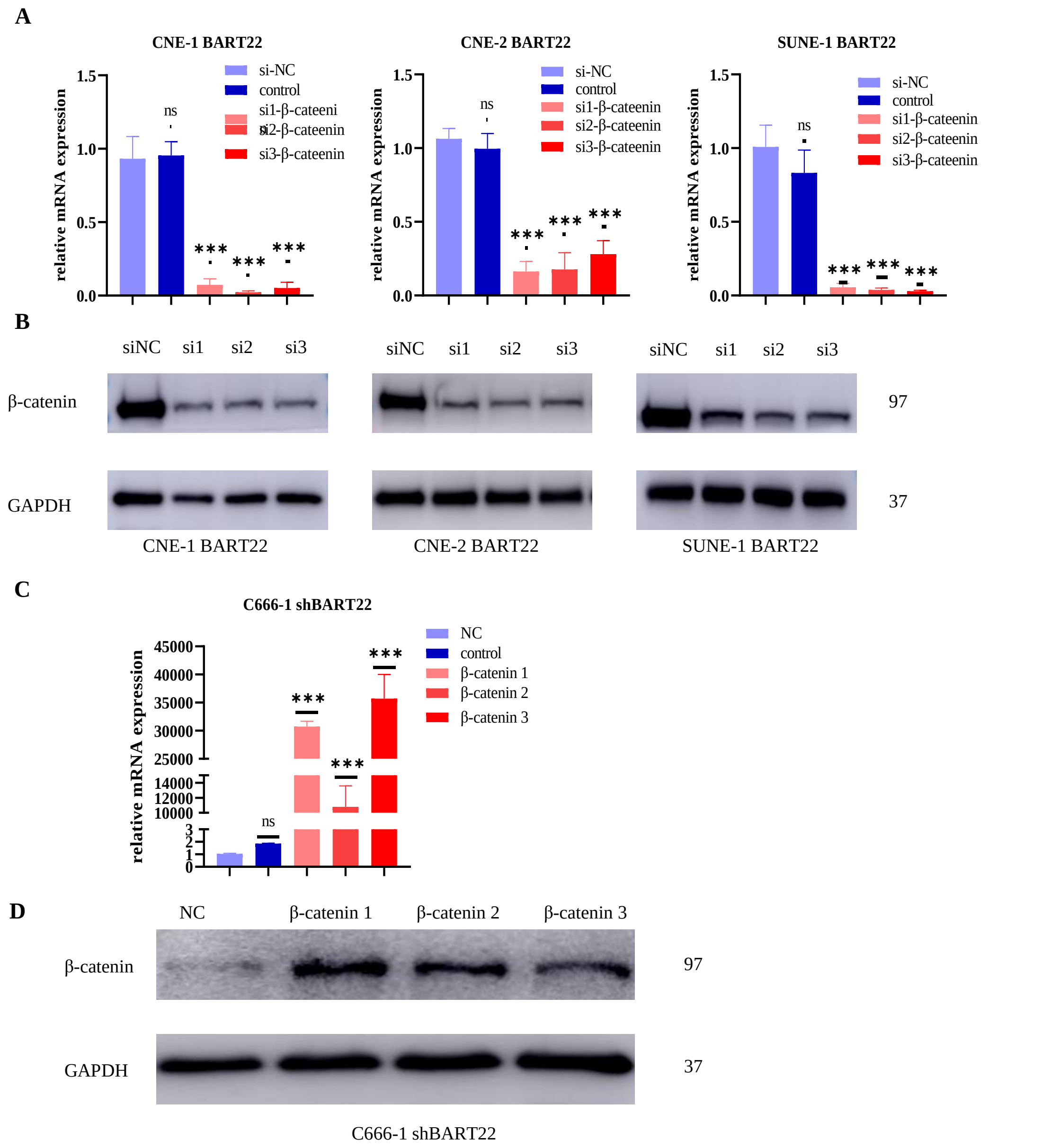

Supplementary Figure 6. The expression of β-catenin mRNA in each cell line after interference or overexpression. A-B. Relative expression of β-catenin mRNA was measured by qRT-PCR and WB in CNE-1 BATRT22, CNE-2 BART22 and SNUE-1 BART22 cells transfected with si-β-catenin. C-D. Relative expression of β-catenin mRNA was measured by qRT-PCR and WB in C666-1 shBATRT22 cells transfected with β-catenin overexpression plasmid.

Supplementary 7

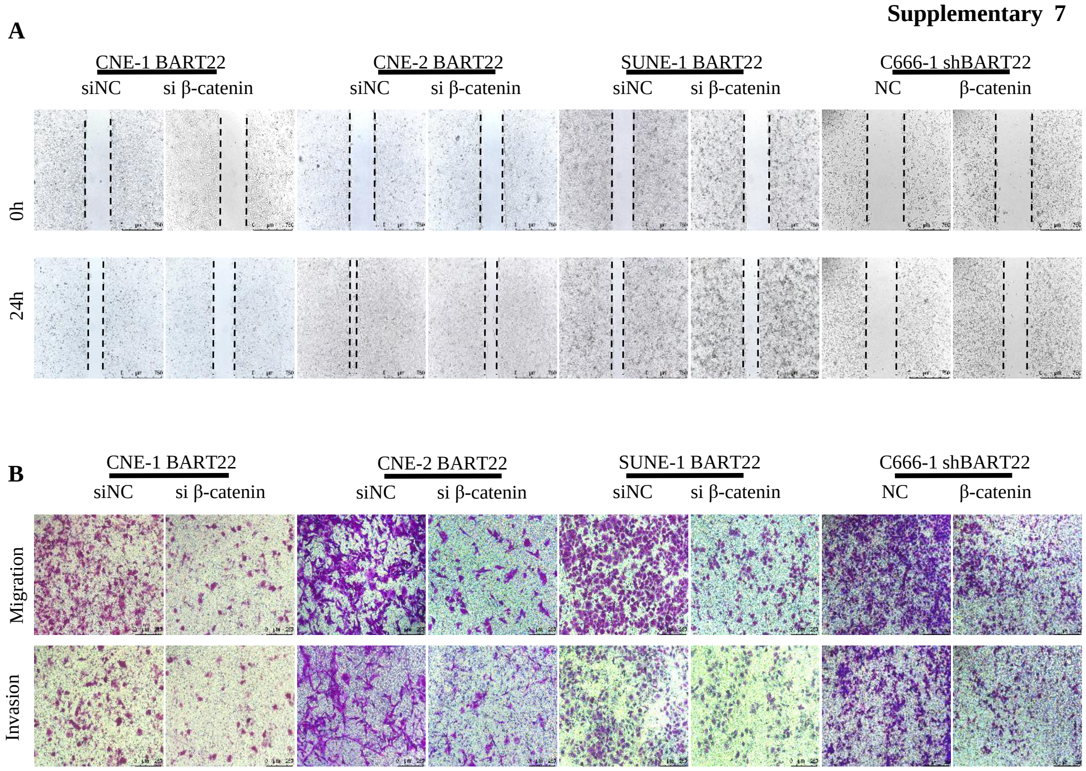

Supplementary Figure 7. EBV-miR-BART22 induces NPC cell metastasis by activating the β-catenin. A. Cells were treated with β-catenin siRNA and plasmid β-catenin for 72h Representative images and quantification of the wound-healing assay in CNE-1 BART22, CNE-2 BART22, SUNE-1 BART22 and C666-1 shBART22 cells. B. Representative images and quantification of migration and invasion assays in CNE-1 BART22, CNE-2 BART22, SUNE-1 BART22 and C666-1 shBART22 cells.

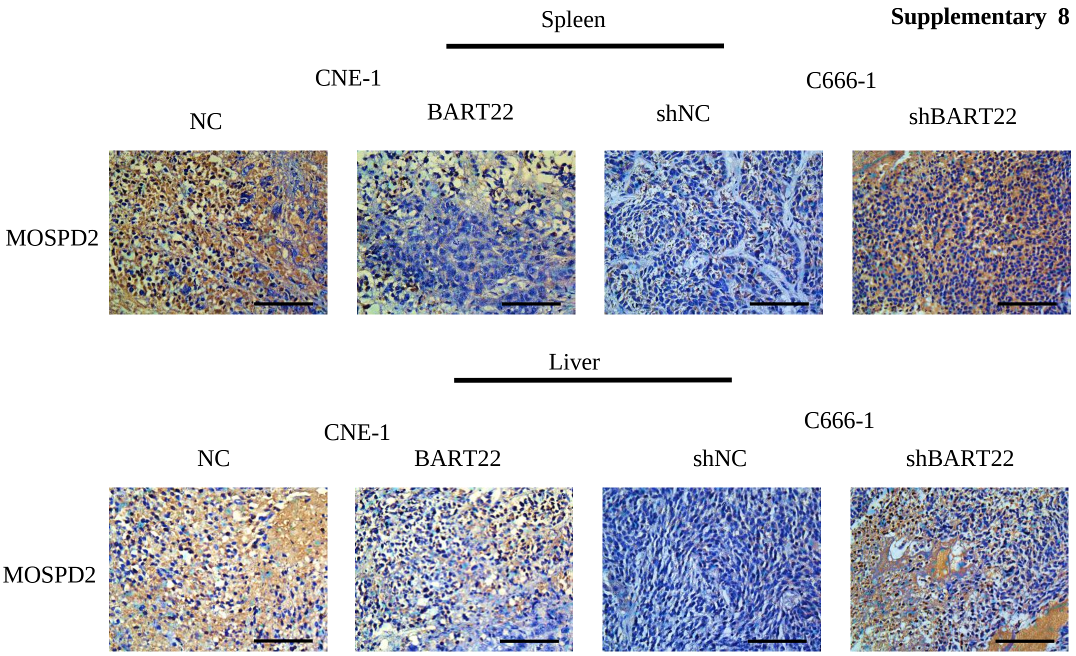

Supplementary Figure 8. MOSPD2 is a direct target of EBV-miR-BART22. IHC analysis of MOSPD2 in spleen tissues and liver metastasis tissues. Representative images are shown

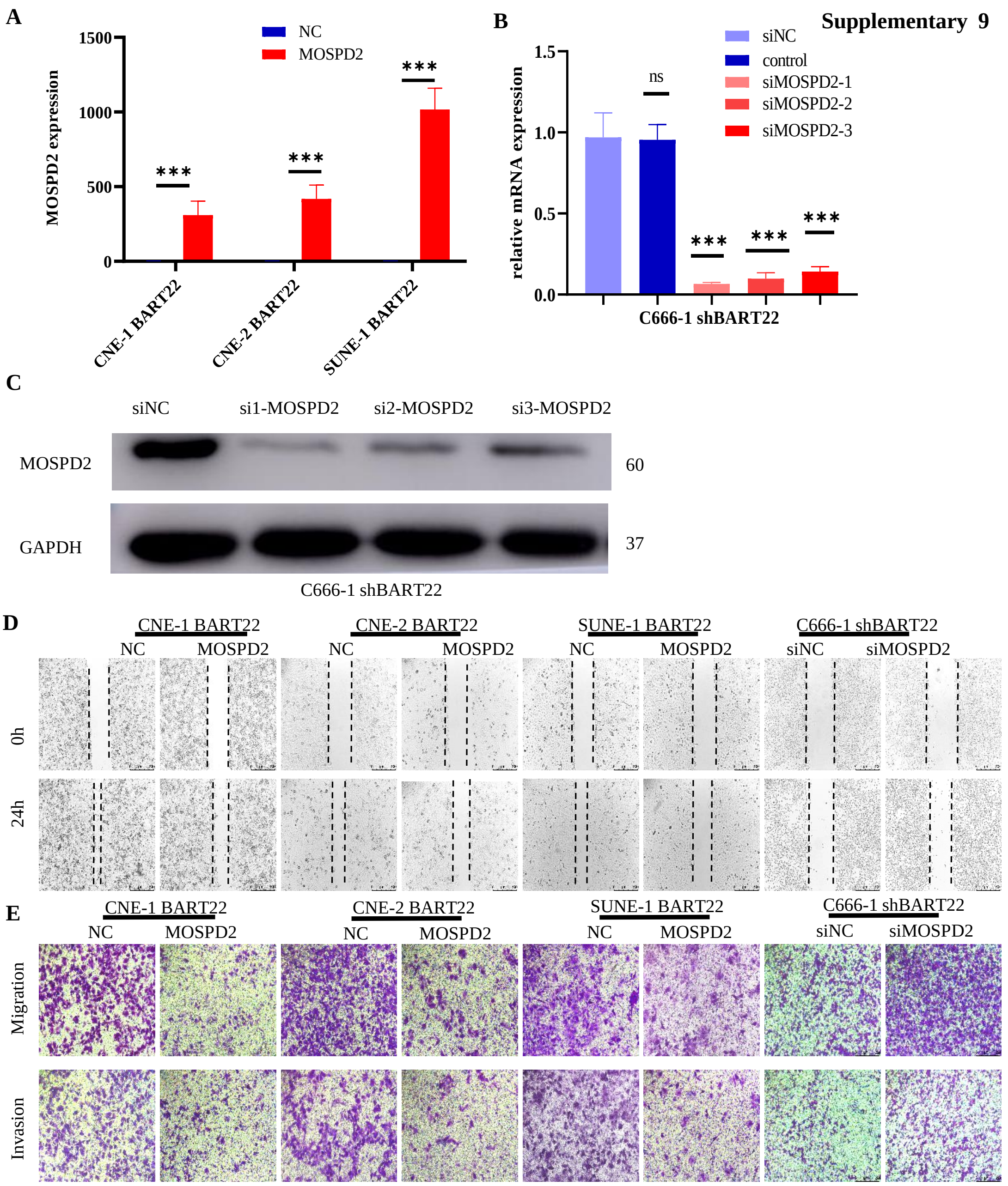

Supplementary Figure 9. The expression of MOSPD2 mRNA in each cell line after interference or overexpression. A-B. Relative expression of MOSPD2 mRNA was measured by qRT-PCR in NPC cells transfected with MOSPD2 overexpression plasmid and si-MOSPD2. C. Relative expression of MOSPD2 mRNA was measured by WB in C666-1 shBART22 cells transfected with si-MOSPD2. D. Cells were treated with MOSPD2 plasmid β-catenin and siRNA for 72h Representative images and quantification of the wound-healing assay in CNE-1 BART22, CNE-2 BART22, SUNE-1 BART22 and C666-1 shBART22 cells. E. Representative images and quantification of migration and invasion assays in CNE-1 BART22, CNE-2 BART22, SUNE-1 BART22 and C666-1 shBART22 cells.

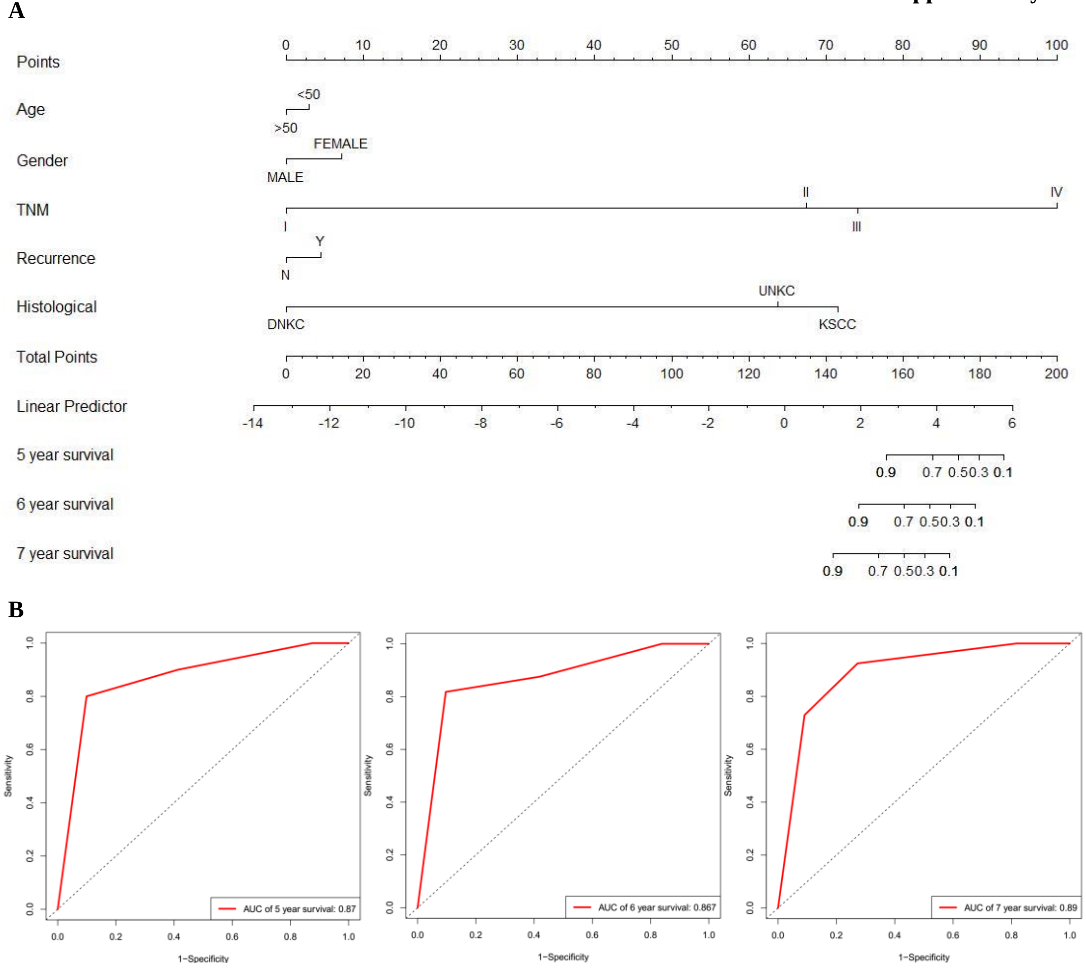

Supplementary Figure 10. A nomogram prediction model was constructed based on the TNM in NPC. A. Nomogram system for the 5-, 6- and 7- year survival rates prediction. The nomogram prediction system was a novel model to estimate OS based on related factors (TNM, Recurrence, Histological type), patient-specific factors (age, gender). B. ROC curves of the 5-, 6- and 7-year nomograms of NPC patients.
