# Supplementary material for: Epstein–Barr virus-encoded microRNA BART22 serves as novel biomarkers and drives malignant transformation of nasopharyngeal carcinoma
Source: Cell Death Dis. 2022 Jul 30;13(7):664. doi: 10.1038/s41419-022-05107-x (PMC9338958; doi:10.1038/s41419-022-05107-x)
Supplement: Supplementary file 4 — Supplementary Table [file 41419_2022_5107_MOESM4_ESM.pdf]

Supplementary Table 1. Association between expression of EBV-miR-BART-22 and main clinical characteristics in 132 NPC patients.

Characteristic		All cases	BART22		Chi-square	*p* value
			low	high		
All cases		132	70	62		
Age	<50	74	39	35	0.007	0.932
	≥50	58	31	27		
Gender	Female	31	15	16	0.351	0.554
	Male	101	55	46		
Clinical stage	I-II	72	50	22	17.134	**<0.001**
	III-IV	60	20	40		
T stage	T1-2	114	66	48	7.942	**0.005**
	T3-4	18	4	14		
N stage	N0	36	23	13	2.343	0.126
	N+	96	47	49		
M stage	0	128	70	58	4.657	**0.031**
	1	4	0	4		
Recurrence	N	75	55	20	28.744	**<0.001**
	Y	57	15	42		
Histological type	UNKC	115	59	56	7.191	**0.027**
	KSCC	3	0	3		
	DNKC	14	11	3		

Supplementary Table 2. Univariate and multivariate Cox hazards analysis of different parameters for OS in 132 NPC patients

Clinical variables	Univariate analysis		*p*	Multivariate analysis		*p*
	HR	95% CI		HR	95% CI	
Age (< 50 vs. ≥50)	0.833	0.356-1.951	0.674	0.699	0.258-1.892	0.481
Gender (Female vs. Male)	1.065	0.393-2.888	0.902	2.518	0.798-7.946	0.115
Clinical stage (I / II vs. III/IV)	13.825	3.230-59.172	**<0.001**	4.071	0.699-23.715	0.118
T stage (T1/2 vs. T3/4)	1.376	0.465-4.066	0.564	0.261	0.075-0.911	**0.035**
N stage (N0 vs. N+)	8.277	1.113-61.541	**0.039**	2.262	0.208-24.586	0.503
M stage (0vs. 1)	14.539	4.755-44.455	**<0.001**	18.380	3.434-98.375	**0.001**
BART22 (low vs. high)	0.032	0.004-0.240	**0.001**	0.117	0.014-0.980	**0.048**
Recurrence (N vs. Y)	9.147	3.014-27.759	**<0.001**	10.595	2.336-48.063	0.002
Histological type (UNKC vs. KSCC vs. DNKC)	0.368	0.084-1.614	0.185	1.123	0.226-5.593	0.887

Supplementary Table 3. GSE54161 miRNAs differentialy expressed in EBV-positive NPC cells versus EBV negative

miRNA	logFC	adj.P.Val
ebv-miR-BART10-3p	15.33012	0.010102
ebv-miR-BART3-3p	12.55688	0.010102
ebv-miR-BART2-5p	12.87449	0.010102
ebv-miR-BART22	11.8087	0.010102
ebv-miR-BART7-3p	10.95766	0.010102
hsa-miR-100-5p	-12.3542	0.010102
ebv-miR-BART19-3p	10.66885	0.010102
hsa-miR-137	-11.1351	0.010102
ebv-miR-BART6-5p	10.18602	0.010102
ebv-miR-BART8-5p	10.18363	0.010102
hsa-miR-100-3p	-10.7784	0.010102
hsa-miR-155-5p	-9.5631	0.010102
ebv-miR-BART15	11.73335	0.010102
hsa-miR-452-5p	-9.48461	0.010102
hsa-miR-542-5p	-8.95761	0.010102
ebv-miR-BART9-3p	9.361924	0.010102
hsa-let-7a-2-3p	-9.64271	0.010102
ebv-miR-BART5-5p	10.18354	0.010102
ebv-miR-BART1-5p	8.817892	0.010102
ebv-miR-BART6-3p	8.536544	0.010102
hsa-miR-224-5p	-9.12398	0.010102
ebv-miR-BART8-3p	9.451704	0.010102
hsa-miR-450a-5p	-8.55174	0.010102
hsa-miR-125b-1-3p	-8.30123	0.010102
ebv-miR-BART4-5p	7.989409	0.010102
hsa-miR-874	-8.08222	0.01085
ebv-miR-BART16	9.243048	0.01085
ebv-miR-BART12	7.293153	0.012212
hsa-miR-654-3p	-8.88776	0.012212
ebv-miR-BART17-5p	7.371781	0.012226
ebv-miR-BART14-3p	8.523587	0.012673
ebv-miR-BART17-3p	7.893777	0.012673
ebv-miR-BART11-5p	6.838047	0.014344
hsa-miR-299-3p	-7.26172	0.015399
hsa-miR-503-5p	-10.8049	0.017138
hsa-miR-424-3p	-9.75331	0.017138
hsa-miR-381-3p	-9.98968	0.017138
ebv-miR-BART7-5p	5.942415	0.018771
hsa-miR-376b-3p	-9.29695	0.019367
ebv-miR-BART13-5p	6.35996	0.021829
ebv-miR-BART9-5p	7.319401	0.024145

ebv-miR-BART13-3p	7.683579	0.024949
ebv-miR-BART21-5p	6.09342	0.026434
hsa-miR-411-5p	-6.1714	0.035442
hsa-miR-369-5p	-4.96439	0.035869
hsa-miR-3182	-6.24521	0.036654
hsa-miR-379-5p	-4.72277	0.037006
hsa-miR-196b-5p	-7.79863	0.037448
ebv-miR-BART1-3p	8.320142	0.037448
hsa-miR-377-3p	-7.15295	0.03876
hsa-miR-1271-5p	-6.23645	0.039959
ebv-miR-BART3-5p	6.687685	0.041946
hsa-miR-584-5p	-7.56604	0.043149
hsa-miR-1271-3p	-8.62264	0.048257

Supplementary Table 4. GSE118720 miRNAs differentialy expressed in NPC versus control

miRNA	logFC	adj.P.Val
hsa-miR-615-3p	5.984377	0.001723
hsa-miR-3150b-3p	-3.59196	0.001723
hsa-miR-4464	-3.38796	0.002174
hsa-miR-142-3p	-3.27024	0.002174
hsa-miR-140-5p	-2.37052	0.002174
hsa-miR-3653-5p	-2.87386	0.002886
hsa-miR-142-5p	-3.38061	0.002886
hsa-miR-363-3p	-3.45183	0.002886
ebv-miR-BART3-3p	7.242434	0.002886
hsa-miR-342-3p	-2.50522	0.002905
hsa-miR-29a-3p	-2.03025	0.002905
ebv-miR-BART18-5p	8.487653	0.003293
hsa-miR-3687	3.244714	0.003293
ebv-miR-BART5-5p	8.364382	0.003293
hsa-miR-766-5p	-2.64655	0.003293
ebv-miR-BART14-5p	8.148858	0.003293
ebv-miR-BART2-5p	9.004737	0.003293
hsa-miR-6744-5p	-2.28466	0.003293
ebv-miR-BART18-3p	8.656934	0.003293
ebv-miR-BART17-3p	8.815794	0.003293
hsa-miR-140-3p	-2.02675	0.003293
hsa-miR-29c-3p	-2.77503	0.003293
ebv-miR-BART4-3p	7.076013	0.003335
ebv-miR-BART8-5p	8.985978	0.003335
ebv-miR-BART6-3p	8.625986	0.003335
ebv-miR-BART14-3p	9.599232	0.003335
ebv-miR-BART1-3p	8.228078	0.003335
ebv-miR-BART7-3p	8.872166	0.003335
ebv-miR-BART22	9.036732	0.003335
ebv-miR-BART1-5p	8.98555	0.003335
ebv-miR-BART19-3p	8.706958	0.003488
ebv-miR-BART7-5p	8.520166	0.003539
ebv-miR-BART10-3p	8.481098	0.003539
hsa-miR-486-3p	-3.12425	0.003539
ebv-miR-BART11-3p	7.989942	0.003539
hsa-miR-29b-3p	-2.54817	0.003539
hsa-miR-342-5p	-3.14818	0.003539
ebv-miR-BART9-5p	8.784317	0.003568
ebv-miR-BART16	8.694536	0.00358
ebv-miR-BART13-5p	8.356647	0.003787
hsa-miR-874-3p	-2.04613	0.003846

hsa-miR-4491	-3.03866	0.003964
ebv-miR-BART4-5p	8.035378	0.003964
ebv-miR-BART8-3p	8.61807	0.003964
ebv-miR-BART6-5p	8.003825	0.00413
ebv-miR-BART3-5p	8.507031	0.004206
hsa-miR-1273h-5p	-2.8587	0.004206
ebv-miR-BART9-3p	8.366798	0.004231
ebv-miR-BART13-3p	8.215687	0.004231
hsa-miR-5196-3p	-2.49611	0.005262
ebv-miR-BART2-3p	6.977228	0.005432
ebv-miR-BART5-3p	5.3908	0.005432
hsa-miR-150-3p	-3.79439	0.005833
ebv-miR-BART11-5p	7.949946	0.006023
hsa-miR-4679	-2.85967	0.006319
ebv-miR-BART21-3p	6.598511	0.006319
ebv-miR-BART21-5p	6.93578	0.006584
ebv-miR-BART12	7.313377	0.006708
hsa-miR-766-3p	-2.3321	0.006708
hsa-miR-6843-3p	-2.58544	0.006708
hsa-miR-1273h-3p	-3.2325	0.007114
ebv-miR-BART10-5p	6.085548	0.007278
hsa-miR-196b-5p	2.649834	0.007456
hsa-miR-6504-5p	-2.49134	0.007484
hsa-miR-6842-5p	-2.07923	0.007592
ebv-miR-BART15	4.730404	0.007648
hsa-miR-20b-5p	-2.84847	0.007648
hsa-miR-150-5p	-4.15337	0.007693
hsa-miR-106a-5p	-2.08103	0.008168
hsa-miR-34b-5p	-4.27265	0.00967
hsa-miR-1275	-2.37289	0.010454
hsa-miR-642a-3p	-2.16978	0.010702
hsa-miR-504-5p	-2.25442	0.011031
hsa-miR-625-5p	-2.24862	0.011263
hsa-miR-548ah-5p	2.33869	0.012754
hsa-miR-34c-5p	-4.83058	0.013298
hsa-miR-138-1-3p	-2.24966	0.013943
hsa-miR-1976	-2.14883	0.013968
hsa-miR-363-5p	-3.47719	0.013971
hsa-miR-338-3p	-2.13299	0.014243
hsa-miR-2110	-2.39885	0.014243
hsa-miR-223-5p	-2.34196	0.014791
hsa-miR-20b-3p	-2.45299	0.017625
hsa-miR-129-1-3p	-2.16887	0.018775
hsa-miR-1277-3p	-2.04687	0.019505

hsa-miR-1247-5p	-2.37422	0.01975
hsa-miR-196a-5p	2.579278	0.01975
hsa-miR-3202	-2.4472	0.020592
hsa-miR-329-3p	2.274984	0.021
hsa-miR-548p	2.162232	0.024943
hsa-miR-4536-5p	-2.51778	0.026281
hsa-miR-449a	-4.67477	0.026281
hsa-miR-548ah-3p	2.317265	0.028163
hsa-miR-138-5p	-2.10987	0.02907
hsa-miR-135b-3p	2.827894	0.03143
hsa-miR-205-3p	2.711741	0.033387
ebv-miR-BART20-3p	3.61767	0.033474
hsa-miR-34b-3p	-4.07184	0.033474
hsa-miR-577	-2.17481	0.03416
hsa-miR-299-3p	2.281425	0.03416
hsa-miR-1255a	-2.51936	0.03416
hsa-miR-190b	-3.09957	0.03864
ebv-miR-BART20-5p	2.955391	0.040777
ebv-miR-BART17-5p	6.782239	0.042499
hsa-miR-135a-5p	-3.04726	0.044367
hsa-miR-493-3p	2.526653	0.048629

Supplementary Table 5.The sequences of PCR primers

EBV-miR-BART-22 forward	5′-TTACAAAGTCATGGTCTAGTAGT-3′
U6 forward	5′-TGCGGGTGCTCGCTTCGGCAGC-3′
GAPDH forward	5′-GGAGCGAGATCCCTCCAAAAT-3′
GAPDH reverse	5′-GGCTGTTGTCATACTTCTCATGG-3′
β-catenin forward	5′-TCCCTGAGACGCTAGATGAGG-3′
β-catenin reverse	5′-CGTTTAGCAGTT TTGTCAGCTC-3′
MOSPD2 forwanrd	5′-TGTGACCCGGGTGCATC-3′
MOSPD2 reverse	5′-TCCATTTCTGCAGCCATTATCA-3′
PUM2 forwanrd	5′-TTCTCAGCAGGCCTTGCTC-3′
PUM2 reverse	5′-GGTGGAACCACTGCTGGAC-3′
ZBTB38 forwanrd	5′-TGTCTTGAAGTGAGGCTCTGCTG-3′
ZBTB38 reverse	5′-AGCAAGCCTTGTGGACCAAAC-3′
RAP2C forwanrd	5′-AGGTAGTGGTGTTAGGGAGC-3′
RAP2C reverse	5′-GCGGTGTCCAGAATTTCCAG-3′
RAB11A forwanrd	5′-CAGCAGGGCAAGAGCGATA-3′
RAB11A reverse	5′-AGCCATCGCTCTACATTTTCA-3′
ZBTB44 forwanrd	5′-GAAGAAGATGTCCGGGTCAA-3′
ZBTB44 reverse	5′-AGTGTAGGCAAGCCCTCAGA-3′
OSBPL8 forwanrd	5′-AGACCAAAGTGAAACATCGCACTC-3′
OSBPL8 reverse	5′-TCAATGGAACTCTGAATGTCTCCC-3′
CPEB4 forwanrd	5′-CAACCCAACCCTTGACATCT-3′
CPEB4 reverse	5′-ACCGTTATTAGCCGAAGCAG-3′
SMAD5 forwanrd	5′-CCAGCAGTAAAGCGATTGTTGG-3′
SMAD5 reverse	5′-GGGGTAAGCCTTTTCTGTGAG-3′
CDH7 forwanrd	5′-CGCCTCCCATGAAGGATCTAC-3′
CDH7 reverse	5′-CTTTTGGGCCTTCTTCCTCTTC-3′
