# Supplementary material for: Epstein–Barr virus-encoded microRNA BART22 serves as novel biomarkers and drives malignant transformation of nasopharyngeal carcinoma
Source: Cell Death Dis. 2022 Jul 30;13(7):664. doi: 10.1038/s41419-022-05107-x (PMC9338958; doi:10.1038/s41419-022-05107-x)
Supplement: Supplementary file 5 — Animal Studies Ethics Committee Approval [file 41419_2022_5107_MOESM5_ESM.pdf]

华南理工大学
South China University of Technology

华南理工大学

实验动物伦理审查意见表

<table>
<tr><td rowspan="2">申请人填写处</td><td>项目名称</td><td colspan="4">EBV-miR-BART22 通过激活 Wnt/β-catenin 信号通路促进鼻咽癌转移的作用与机制</td></tr>
<tr><td>申请人</td><td colspan="4">张婷</td></tr>
<tr><td rowspan="13">实验动物伦理委员会填写处</td><td>受理编号</td><td colspan="4">2019030</td></tr>
<tr><td>审查形式</td><td>☐会议　☑通讯</td><td>审查时间</td><td colspan="2">2019 年 7 月 12 日</td></tr>
<tr><td colspan="5">审 查 内 容</td></tr>
<tr><td>审查项目</td><td colspan="4">审查项目</td></tr>
<tr><td>参与实验人员资质</td><td colspan="4">☐ 全部持证　☑ 部分持证　☐ 全部无证</td></tr>
<tr><td>研究项目的重要性</td><td colspan="4">☐ 很重要　☑ 有价值　☐ 有限或无法评估</td></tr>
<tr><td>达到研究目的可能性</td><td colspan="4">☐ 很可能　☑ 可能　☐ 有限或无法评估</td></tr>
<tr><td>动物种类和数量选择的合理性</td><td colspan="4">☐ 理由充分　☑ 较合理　☐ 缺乏有效证据</td></tr>
<tr><td>动物疼痛和痛苦控制措施</td><td colspan="4">☑ 措施得当　☐ 措施尚可　☐ 措施不足</td></tr>
<tr><td>动物术后护理及安乐死处理措施</td><td colspan="4">☑ 措施得当　☐ 措施尚可　☐ 措施不足</td></tr>
<tr><td colspan="5">审 查 意 见</td></tr>
<tr><td colspan="5">综合评价：
该项目的动物实验方案符合实验动物福利和伦理的要求与规定，同意开展研究。

☑建议批准实验；☐ 调整方案后，可以进行实验；☐ 建议不予批准</td></tr>
<tr><td colspan="5">华南理工大学实验动物伦理委员会（公章）</td></tr>
</table>
