# Supplementary material for: Epstein–Barr virus-encoded microRNA BART22 serves as novel biomarkers and drives malignant transformation of nasopharyngeal carcinoma
Source: Cell Death Dis. 2022 Jul 30;13(7):664. doi: 10.1038/s41419-022-05107-x (PMC9338958; doi:10.1038/s41419-022-05107-x)
Supplement: Supplementary file 6 — Ethics Committee Approval and Patient Consent [file 41419_2022_5107_MOESM6_ESM.pdf]

	**上海芯超生物科技有限公司**<br>Shanghai Outdo Biotech Company	Barcode
	伦理委员会审核意见书	编号/Ctl No. YB M-05-02<br>版本/Version 2.0

项目名称	EBV-miR-BART22 促进鼻咽癌转移的作用与机制		
项目来源	国家自然科学基金	项目承担单位	广州医科大学
项目编号	8170110892	项目负责人	张婷
审核结果	参加伦理委员会会议 7 人，投票结果：<br><br>A）同意 7 票<br>B）修正后再审查 ___ 票<br>C）不同意 ___ 票<br>D）暂停或终止 ___ 票<br><br>伦理委员会审查意见：<br><br>申报内容经审查，符合伦理要求，同意开展研究。		
	记录人签名：<br>陈红圭<br><br>2019 年 12 月 6 日	主任委员签名：<br>（伦理委员会盖章）<br>上海芯超生物科技有限公司 伦理委员会<br><br>年 月 日	

保存期限：五年

	**上海芯超生物科技有限公司**<br>Shanghai Outdo Biotech Company	Barcode
	样本使用伦理审查申请表	编号/Ctl No.: YB M-05-01<br>版本/Version: 2.0

项目名称:	EBV-miR-BART22 促进鼻咽癌转移的作用与机制	项目编号:	8170110892
项目来源:	国家自然科学基金	资金来源:	国家自然科学基金
样本名称:	132 点组织芯片 HNasN132Su01	样本数量:	共 132 点
申请人:	陈醉	联系方式:	13507410575
研究者 1:	陈醉　职称：无	研究者 2:	职称:
项目承担单位:	广州医科大学		
项目负责人:	张婷	联系方式:	13416108266
拟研究时间:	2017 年 3 月 21 日 至 2021 年 12 月 10 日		
研究设计	一、 请阐述该样本用于研究的描述（包括目标和假设）:<br>通过检测鼻咽癌组织芯片，发现不同时期的病人标本中的 EBV-miR-BART22 含量是否存在差异。EBV-miR-BART22 是否具有抑制鼻咽癌细胞 EMT 的作用。<br>二、 项目研究的方法:<br>用原位杂交方法检测该样本中配对鼻咽癌癌组织和癌旁组织 EBV-miR-BART22 的表达水平高低，得出 H-score，用 t 检验比较两者 H-score 均值差异；用 ROC 曲线结合生存状态得出 H-score 的 cut-off 值，将 EBV-miR-BART22 分为高表达和低表达组，用 KM 法比较两组生存差异。<br>三、备注:		
说　明	为有效利用宝贵的样本资源，您需向样本库提供一份简要的研究设计（如上）和所要求的资料，您要保证您将合法地使用样本，并进行您的上述研究，您需承诺在您的研究成果和文章上注明样本来源于：<br>上海芯超•生物样本库<br>申请程序：下载申请表----填写申请表----负责人签字（盖章）----样本库伦理委员会审核		

申请人签字:<br>陈醉<br>2019 年 12 月 3 日	申报部门意见:<br>部门负责人签字:<br>2019 年 12 月 3 日	样本库审核意见:<br>样本库负责人签字:<br>2019 年 12 月 3 日

保存期限：五年
